# Effect of Different Antibiotic Chemotherapies on *Pseudomonas aeruginosa* Infection *In Vitro* of Primary Human Corneal Fibroblast Cells

**DOI:** 10.3389/fmicb.2017.01614

**Published:** 2017-08-22

**Authors:** Maria del Mar Cendra, Myron Christodoulides, Parwez Hossain

**Affiliations:** ^1^Molecular Microbiology, Academic Unit of Clinical and Experimental Sciences, Faculty of Medicine, University of Southampton Southampton, United Kingdom; ^2^Eye Unit, Academic Unit of Clinical and Experimental Sciences, University Hospital Southampton NHS Foundation Trust, Southampton General Hospital Southampton, United Kingdom

**Keywords:** *Pseudomonas aeruginosa*, bacterial keratitis, antibiotic chemotherapy, intracellular persistence, corneal diseases, *in vitro* model

## Abstract

*Pseudomonas aeruginosa* is a major cause of bacterial keratitis (BK) worldwide. Inappropriate or non-optimal antibiotic chemotherapy can lead to corneal perforation and rapid sight loss. In this study, we tested the hypothesis that *P. aeruginosa* strain PAO1 invades primary human corneal fibroblasts (hCFs) *in vitro* and persists intracellularly, despite chemotherapy with antibiotics used commonly to treat BK. In rank order, ciprofloxacin, levofloxacin and polymyxin B showed the highest activity against planktonic PAO1 growth (100% inhibitory concentration ≤10 μg/mL; 50% inhibitory concentration ≤1 μg/mL), followed by gentamicin and ofloxacin (100% inhibitory concentration ≤50 μg/mL; 50% inhibitory concentration ≤10 μg/mL). These bactericidal antibiotics (50–200 μg/mL concentrations) all killed PAO1 in the extracellular environment of infected hCF monolayers. By contrast, the bactericidal antibiotic cefuroxime and the bacteriostatic antibiotic chloramphenicol failed to sterilize both PAO1 broth cultures, even at a concentration of ≥200 μg/mL) and infected hCF monolayers. Statistically, all antibiotics were able to prevent LDH release from PAO1-infected hCF monolayers at both concentrations tested. Intracellular *Pseudomonas* were significantly reduced (>99%, *P* < 0.05) following treatment with ciprofloxacin, levofloxacin and ofloxacin, whereas gentamicin, polymyxin B and cefuroxime failed to clear intracellular bacteria over 24 h. Intracellular *Pseudomonas* infection was resistant to chloramphenicol, with hCF death observed by 9 h. Eventual growth of remaining intracellular *Pseudomonas* was observed in hCF after removal of all antibiotics, resulting in re-infection cycles and cell death by 48 h. All of the antibiotics reduced significantly (*P* < 0.05) IL-1β secretion by hCF infected with a Multiplicity Of Infection (MOI) = 1 of PAO1. With higher MOI, no pro-inflammatory effects were observed with antibiotic treatment, expect with polymyxin B and ofloxacin, which induced significant increased IL-1β secretion (*P* < 0.001). The findings from our study demonstrated that bactericidal and bacteriostatic antibiotics, routinely used to treat BK, failed to eradicate *Pseudomonas* infection of hCFs *in vitro* and that their bactericidal efficacies were influenced by the cellular location of the organism.

## Introduction

*Pseudomonas aeruginosa* is the leading cause of bacterial keratitis (BK) associated with contact lens wear (Shah et al., 2011; Stapleton and Carnt, 2012). Like other *Pseudomonas* infections, untreated or inappropriately treated BK can lead to rapid progression with adverse outcomes from rapid and permanent sight loss from corneal opacification/perforation (Donzis, 1998; Sy et al., 2012; Vazirani et al., 2015). A healthy cornea is inherently resistant to microbial infections but when the epithelium is breached, e.g., following surgery, trauma or contact lens wear, microbes can penetrate and gain access to the stroma (Taube et al., 2015). Infection can become established in the corneal stroma and is characterized by bacterial growth and dissemination (Badenoch and Coster, 1989; O’Brien, 2003; Ong and Corbett, 2015). In response to bacteria-induced injury and inflammation, resident stromal keratocytes can transform into corneal fibroblasts and exacerbate inflammation by secreting pro-inflammatory mediators and recruiting leukocytes (Smith et al., 1997; Wong et al., 2011).

Treatment of *Pseudomonas* keratitis requires disinfection of the eye with effective antibiotics. Some pathogens, including *Pseudomonas* spp., can adapt their lifestyle to avoid innate immune defenses and antimicrobial activities, e.g., by penetrating inside eukaryotic host cells or by forming biofilms (Carryn et al., 2003; del Pozo and Patel, 2007; Lebeaux et al., 2014; Rybtke et al., 2015). Cellular localization of the bacteria and differences in the pharmacodynamic properties of antibiotics used routinely, may have a high impact on the treatment and successful chemotherapy can be difficult if the required inhibitory antibiotic dosages are not achieved (Carryn et al., 2003). Failure to achieve bacteriological eradication may be a factor in the persistence of *Pseudomonas* infection within the tissue as a result of the invasive genotype (Shen et al., 2015) and recurrent infection despite antibiotic treatment (Ti et al., 2007). Additionally, over-stimulation of the inflammatory response is a common issue during BK treatment (Taube et al., 2015). It is not known also if different antibiotic regimens have inherent inflammatory effects that may impact on the corneal healing response. Corticosteroids are known to possess anti-inflammatory properties, but their use is not recommended until infection is cleared (Gokhale, 2008).

A large literature reports animal models for investigating bacterial factors and host cellular responses in BK (Marquart, 2011). *P. aeruginosa* has been reported to invade corneal epithelial cells of the mouse and rabbit *in vivo* (Fleiszig et al., 1996; Lee et al., 2003a; Yamamoto et al., 2006). In these studies, an inverse correlation was observed between cytotoxicity and bacterial invasion (Fleiszig et al., 1996). Furthermore, the invasive or cytotoxic background of the *P. aeruginosa* strain appeared to affect keratitis treatment of murine models *in vivo* (Lee et al., 2003b). The aminoglycoside antibiotic tobramycin, which poorly penetrates eukaryotic cell membranes, was found to be less effective at eradicating invasive *P. aeruginosa* strains in mouse corneas, than the penetrating fluoroquinolone antibiotic ofloxacin (Lee et al., 2003b). In humans, an increase in the number of isolates resistant to fluoroquinolones was observed if *P. aeruginosa* encoded the type three secreted cytotoxin gene *exoU* (Borkar et al., 2014). Patients with ulcers caused by fluoroquinolone-resistant *P. aeruginosa* that expressed *exoU* trended toward poorer visual outcomes than patents with ulcers caused by bacteria that were non-resistant but still expressed *exoU*.

A very large literature exists also on the ability of *P. aeruginosa* to adhere to cultured myeloid and non-myeloid, primary and transformed mammalian cells *in vitro* and in *vivo*, and to subsequently invade (Bucior et al., 2014). For example, *P. aeruginosa* can adhere to and invade non- phagocytic cells such as human A549 lung epithelial cells and Caco-2 colon cells, monocytic THP-1 cells, and also different human primary cells, e.g., epithelial cells from the cornea or nose (Fleiszig et al., 1995; Esen et al., 2001; Ulrich et al., 2005; Kim and Wei, 2007; Ahmed et al., 2014). In addition, the key mechanisms of adherence and invasion, as well as intracellular persistence, pathogen exit and avoidance of innate immunity have been identified along with several of the *Pseudomonas* and host cellular and genetic factors involved (Bleves et al., 2010; Gellatly and Hancock, 2013; Lovewell et al., 2014). The bactericidal activity of different antibiotics against extracellular and intracellular *Pseudomonas* growth also has been investigated in some of these *in vitro* cell culture models (Smith et al., 2000; Ulrich et al., 2005; Kim and Wei, 2007; Buyck et al., 2013). Recently, we reported that *P. aeruginosa* can adhere to human primary corneal fibroblasts (hCFs) *in vitro* and subsequently invade. We also reported that hCFs were a source of IL-1β secretion in response to challenge with *P. aeruginosa in vitro* (Cendra et al., 2017). IL-1β is a pleiotropic cytokine that is involved in several inflammatory processes involving a variety of different eukaryotic cells (Ren and Torres, 2009) and over-stimulated IL-1β expression can contribute to impaired healing (Okamoto et al., 2004; Stapleton et al., 2008). In the current study, we tested the hypothesis that *P. aeruginosa* strain PAO1 invading primary human corneal fibroblasts (hCFs) *in vitro* could persist intracellularly, despite chemotherapy with antibiotics used commonly to treat BK, e.g., fluoroquinolone (ciprofloxacin, levofloxacin, ofloxacin), aminoglycoside (gentamicin), polypeptide (polymyxin B), semisynthetic cephalosporin (cefuroxime) and synthetic bacteriostatic (chloramphenicol) antibiotics. We have screened and compared the pharmacodynamics, antimicrobial and anti-inflammatory activities of these different antibiotic therapies currently available for clinical use in the United Kingdom to treat BK (Tuft and Burton, 2013), and ranked them according to their impact on *Pseudomonas* infection of hCFs *in vitro* and on *Pseudomonas* intracellular persistence.

## Materials and Methods

### Bacteria and Growth Conditions

*Pseudomonas aeruginosa* strain PAO1 (Holloway1C Stanier131) was obtained from the National Collection of Industrial, Food and Marine Bacteria (NCIMB), United Kingdom. PAO1 was grown in Luria-Bertani (LB, Oxoid) broth and on nutrient agar (Oxoid) at 37°C.

### Primary Human Corneal Fibroblast Cells

Corneal epithelium was scraped from corneo-scleral rims of healthy donors and the stromal layer was dissected and digested with collagenase type-1 (1 mg/mL; Life Technologies) for 3 h at 37°C. Digested stroma was cultured in Dulbecco’s Modified Eagle’s Medium (DMEM, Sigma–Aldrich) supplemented with 5% (v/v) decomplemented Fetal Calf Serum (dFCS, Life Technologies), 100 Units/mL of penicillin and 100 Units/mL of streptomycin (Life Technologies) and 0.5 μg/mL of amphotericin B (Lonza) and fibroblasts were characterized as described previously (Wong et al., 2011). The number of cell passages used in this study was kept between 3 and 6 to minimize fibroblast differentiation to myofibroblast, as seen in other studies (Masur et al., 1995; Bargagna-Mohan et al., 2015). Fibroblasts from each donor were kept separate (not pooled) and used for independent experiments. Cells were incubated in a humidified atmosphere containing 5% (v/v) CO_2_ at 37°C.

In experiments to examine the efficacy of different antibiotics against extracellular and intracellular PAO1, hCFs were cultured in antibiotic-free DMEM supplemented with 5% (v/v) dFCS for at least one passage prior to bacterial challenge, in order to ensure the absence of possible traces of antibiotics.

### Ethics Statement

Patients provided written informed consent in accordance with the Declaration of Helsinki to use surplus corneal tissue specimens for research via the NHS Blood Transplant Eye Retrieval Service. Protocols were approved by the NRES Committee South Central - Berkshire 06/Q1602/56.

### Antibiotics and Determination of Activity against *P. aeruginosa* Using a Broth Dilution Assay

The antibiotics chosen for this *in vitro* study were based on those commonly used in United Kingdom clinical practice, namely the bactericidal antibiotics ciprofloxacin (CIP, Sigma–Aldrich), cefuroxime 0.5% (w/v) (CXM, Hampshire Hospitals NHS), polymyxin B (PMB, Sigma–Aldrich), gentamicin (GEN, Sigma–Aldrich), ofloxacin (0.3% w/v) (OFX, Allergan), levofloxacin (0.5% w/v) (LVX, Santen) and the bacteriostatic antibiotic chloramphenicol 0.5% (w/v) (CHL, Bausch and Lomb). A broth dilution assay was done to determine PAO1 susceptibility to each antibiotic. Various concentrations of antibiotic (0.01, 0.1, 1, 10, 50, 100, and 200 μg/mL) were added to equal volumes of LB broth medium with various concentrations of bacteria (∼10^5^, ∼10^6^ and ∼10^7^ Colony Forming Units (CFU)/mL). Absorbance (Optical Density (OD) λ_600_ nm) as a measure of bacterial growth was assessed hourly up to 9 h and then at 24 h of incubation at 37°C with shaking (200 rpm). Comparison of the 24 h OD readings for bacterial growth with and without antibiotics was used to calculate the lowest concentration of antibiotic that completely reduced OD growth by 100% and the concentration of antibiotic that reduced OD growth by 50%.

### *P. aeruginosa* Infection of hCFs

Extracellular and intracellular PAO1 bacteria were quantified in the presence of each antibiotic used at concentrations of 50 and 200 μg/mL. Confluent hCF monolayers were challenged in triplicate in 24-well cell culture plates (Greiner Bio-one, ∼10^5^ hCF/well) with different Multiplicities Of Infection (MOI = 1, ∼10^5^ CFU/ml; MOI = 10, ∼10^6^ CFU/mL and MOI = 100, ∼10^7^ CFU/mL) of PAO1. At given time-points, cell monolayers were washed gently four times with phosphate buffered saline (PBS), pH7.4 and associated PAO1 bacteria were quantified by viable counting on nutrient agar after hCF lysis with a buffer of PBS containing 0.1% (w/v) saponin and 0.1% (v/v) dFCS, as described previously (Hung et al., 2013).

The gentamicin exclusion assay was used to eliminate extracellular bacteria in order to subsequently quantify intracellular bacteria. The assay was done as described previously (Hung et al., 2013), whereby infected cell monolayers are washed with PBS, pH 7.4 and then incubated with a solution of gentamicin (200 μg/mL) for 90 min to kill extracellular bacteria. The monolayers were washed to remove gentamicin and then lysed with a solution of PBS containing saponin (0.1% w/v) and dFCS (0.1% v/v) and the surviving intracellular bacteria enumerated by viable counting on NA.

To quantify the effects of the different antibiotics on survival of intracellular PAO1 bacteria, 1 mL of DMEM containing 50 or 200 μg/mL of the antibiotics listed above was added to hCFs after 3 h of PAO1 infection at the different MOIs, and intracellular PAO1 enumerated after 1.5, 4.5, 7.5, and 24 h of each antibiotic treatment. At these given time points, monolayers were washed four times with PBS, lysed and intracellular bacteria quantified as described above. Extracellular growth of PAO1 during cell monolayer infection with different antibiotic treatments was quantified by viable counting at 0, 1, 3, 6, 9, and 24 h on nutrient agar.

### Quantification of Extracellular IL-1β Cytokine

hCF monolayers were infected for 3 h with PAO1 (MOI = 1, 10, and 100), then treated with antibiotics and IL-1β cytokine was quantified from 9 h antibiotic-treated-hCF supernatants using the Meso Scale Discovery (MSD) electro-chemiluminescence assay (MesoScaleDiagnostics), following the manufacturer’s instructions.

### Cytotoxicity Activity

The cytotoxicity of CIP, CXM, PMB, GEN, OFX, LVX and CHL (50 and 200 μg/mL) alone to hCFs and of PAO1 infection (MOI = 1, 10, and 100) with and without antibiotics, was examined after 0, 1, 3, 6, 9, and 24 h of incubation. Release of lactate dehydrogenase (LDH) was measured using the Pierce LDH Cytotoxicity Assay Kit (Thermo-Scientific) and the percentage cytotoxicity was calculated following the manufacturers’ instructions.

### Statistics

Data were analyzed by one-way ANOVA with Dunnett’s multiple comparison test or by unpaired *t*-test. *P*-values < 0.05 denoted significance.

## Results

### Pharmacodynamics of Antibiotic Chemotherapies for *P. aeruginosa* PAO1 Infection of Primary hCF Cultures *In Vitro*

In the current study, we used an *in vitro* model of primary hCF cells derived from the stroma, to evaluate current antibiotic treatments used in the United Kingdom to treat eye infections. Initially, we used a broth dilution method to calculate the lowest concentration of GEN, CXM, OFX, PMB, CIP, CHL and LVX antibiotics that completely reduced OD growth of *P. aeruginosa* strain PAO1 (tested at various concentrations of ∼10^5^ – ∼10^7^ CFU/mL) by values of 50 and 100%. In rank order, the fluoroquinolones CIP and LVX and polymyxin PMB showed the highest antimicrobial activities with 100% reduction in OD with a concentration ≤10 μg/mL and 50% reduction in OD with a concentration ≤1 μg/mL (**Table 1**). This was followed by GEN and OFX (100% reduction in OD with concentrations ≤50 μg/mL and 50% reduction in OD with concentrations ≤10 μg/mL) and lastly by CXM and CHL treatments, which showed the lowest activities (≥200 μg/mL) and failed to sterilize the PAO1 broth cultures (**Supplementary Figure S1** and Table S1). LB broth could support the planktonic growth of *P. aeruginosa* PAO1 over time in the absence of antibiotics (**Supplementary Figure S2**).

**Table 1:** Effect of antibiotics on the growth of *P. aeruginosa* PAO1, determined with a broth dilution assay.

	CIP		LVX		PMB		GEN		OFX		CXM		CHL	
PAO1 MOI	100%	50%	100%	50%	100%	50%	100%	50%	100%	50%	100%	50%	100%	50%
**1**	> 1; < 10	>0.1; < 1	10	> 1; < 10	10	1	10	> 1; < 10	50	10	>200	200	>200	>200
**10**	10	1	10	> 1; < 10	10	1	50	10	50	10	>200	200	>200	>200
**100**	10	1	10	> 1; < 10	10	1	50	10	50	10	>200	200	>200	>200

*Bacterial growth was recorded by measuring optical density (λ_*600*_nm) over time in a broth dilution assay. The concentrations of antibiotics shown (units = μg/mL) are the lowest concentrations that reduced optical density readings at 24 h completely (100%) and by 50%.*

**FIGURE 1:**
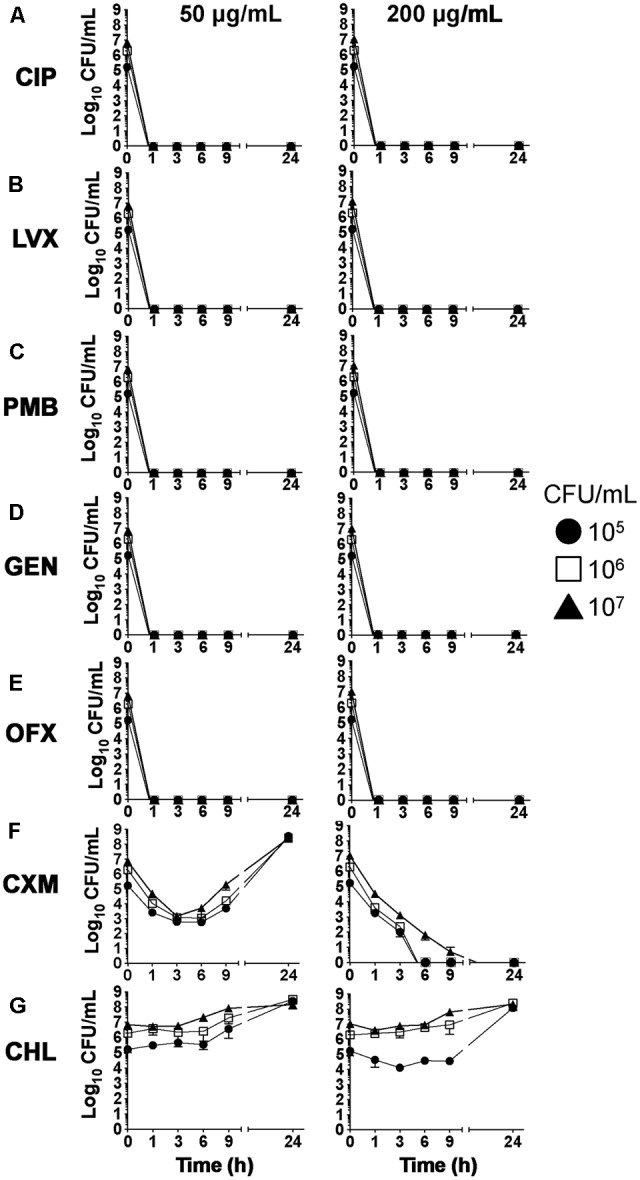
Extracellular *P. aeruginosa* growth during hCF-infection in the presence of antibiotics. hCF monolayers were infected with different concentrations of *P. aeruginosa*, ∼10^5^ CFU/ml (MOI = 1), ∼10^6^ CFU/mL (MOI = 10) and ∼10^7^ CFU/mL (MOI = 100), and treated with the different antibiotics (CIP, LVX, PMB, GEN, OFX, CXM and CHL) at 50 and 200 μg/mL. Bacterial growth (CFU) was quantified from *n* = 3 monolayers at 0, 1, 3, 6, 9, and 24 h. The symbols represent the mean values for CFU/mL of bacterial growth in antibiotic-containing medium from *n* = 3 independent experiments and the error bars the standard error of the means (SEM). A one-way ANOVA with Dunnett’s multiple comparison test was used to compare the reductions in CFU numbers after antibiotic treatment over time with the initial infecting MOIs (CFU at time = 0 h).

Next, we examined the bactericidal ability of the antibiotic panel to control PAO1 extracellular infection of cultured hCF cells. The antibiotic concentrations to be tested were chosen as 50 and 200 μg/mL based on their ability to reduce planktonic growth of PAO1 in broth (**Table 1**). For some of the antibiotics, e.g., CIP, LVX and PMB, these concentrations were significantly higher than the concentrations that could completely inhibit bacterial growth as judged by the broth dilution assay, but could be seen as potentially representing accumulated antibiotic concentrations in clinical use with repeated dosing of infected eye tissue. hCF cultures were infected with PAO1 (MOI = 1, = 10, = 100), antibiotics added at time = 0 h and extracellular growth of the organism quantified (CFU) over time (**Figure 1**). Concentrations of 50 and 200 μg/mL of CIP, LVX, PMB, GEN and OFX killed all PAO1 (>99.99%; *P* < 0.0001) in the extracellular environment by 1 h (**Figure 1A–E**). Treatment with CXM at 50 μg/mL resulted in a >99% reduction of extracellular bacterial CFU by 6 h (*P* < 0.05), but this was not maintained and bacterial CFU increased thereafter to levels similar to bacterial growth observed in PAO1-infected monolayers not treated with antibiotics (≥10^8^–10^9^ CFU/mL; *P* > 0.05, using an unpaired *t*-test; **Supplementary Figure S3**). CXM treatment only killed extracellular PAO1 when used at a concentration of 200 μg/mL (*P* < 0.05 up to 6 h and *P* < 0.001 at 24 h), with sterility occurring between 6–9 h (**Figure 1F**). By contrast, CHL was ineffective at killing PAO1 at both concentrations (**Figure 1G**) (*P* > 0.05), with extracellular CFU of PAO1 increasing over time to levels (≥10^8^ CFU/mL; *P* > 0.05, using an unpaired *t*-test) similarly observed in PAO1-infected monolayers not treated with antibiotics (**Supplementary Figure S3**).

PAO1 infection is cytotoxic to hCFs *in vitro*, with MOI-dependent cell death occurring between 9–24 h, as judged by loss of monolayer integrity and increased release of LDH due to membrane damage (**Supplementary Figure S4A**). We next examined whether the antibiotic treatments protected hCF monolayers from PAO1-induced cell death. In general, antibiotics alone were not cytotoxic to hCFs *in vitro*, except for treatment with PMB, which increased LDH release by 30–40% by 24 h, and with OFX to a lesser extent (∼10% increased LDH release) (**Supplementary Figure S4B**). By visual inspection, PAO1-infected hCF monolayers treated and maintained with the antibiotics CIP, LVX, GEN and PMB were intact after 24 h. OFX-treated hCF cells showed signs of apoptosis by morphology, i.e., cell rounding and shrinkage, although the monolayers were still intact. By contrast, CXM or CHL treatments failed to protect hCF monolayers from PAO1 infection, with cell death and monolayer detachment occurring between 9 and 24 h.

**FIGURE 2:**
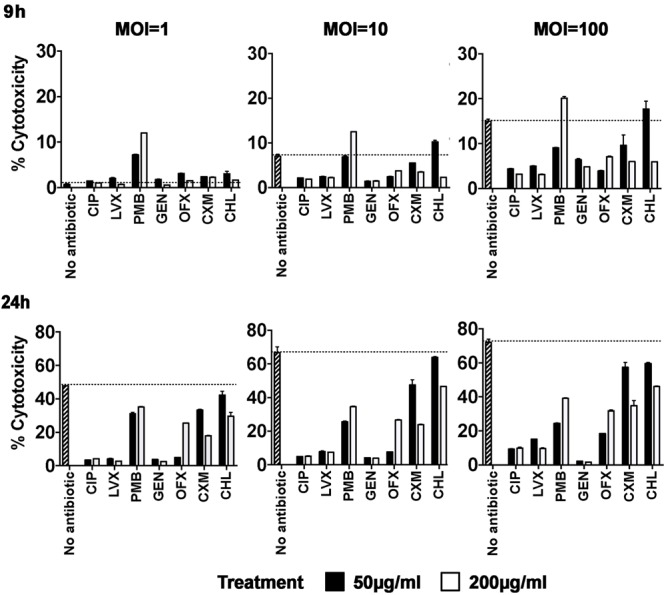
Cellular toxicity induced by *P. aeruginosa* infection of hCFs in the presence of different antibiotics. hCF monolayers were infected with *P. aeruginosa* at MOI = 1, 10, and 100, and CIP, LVX, PMB, GEN, OFX, CXM and CHL (50 and 200 μg/mL) was added to each monolayer at 3 h post initial infection. LDH release was measured from supernatants after 9 and 24 h of each antibiotic treatment and the percentages of cytotoxicity were calculated following the manufacturer’s instructions. Non-antibiotic treated and infected hCF monolayers were used as controls. The columns represent the mean percentage of cytotoxicity measured as LDH release from monolayers from *n* = 3 independent experiments and the error bars represent the standard error of the means (SEM). A one-way ANOVA with Dunnett’s multiple comparison test was used to compare the percentage cytotoxicity levels observed with the antibiotic treatments against the different MOI with the no treatment control, at both 9 h and 24 h.

The protective effect of antibiotics was confirmed by measuring LDH release (**Figure 2**). Monolayers were infected with PAO1 (MOI = 1, 10, 100), antibiotics were added at 3 h and LDH release measured at 9 and 24 h. At a MOI = 1, there was no significant difference at 9 h in the cytotoxicity levels between CIP and GEN concentrations and no antibiotic (*P* > 0.05), whereas cytotoxicity levels were increased marginally following LVX, OFX, CXM, CHL (*P* < 0.05) and significantly with PMB (*P* < 0.0001) treatments. By 24 h, significant reductions in cytotoxicity were observed with CIP, LVX, GEN and OFX (*P* < 0.0001), PMB and CXM (*P* < 0.05) and CHL (*P* < 0.01).

At a MOI = 10, there was a significant reduction by 9 h in percentage cytotoxicity with 50 and 200 μg/mL concentrations of CIP, LVX and GEN (*P* < 0.001) and OFX (*P* < 0.05) and with 200 μg/mL concentration only of CXM (*P* < 0.05). With PMB treatment, there was no significant difference in cytotoxicity levels with the 50 μg/mL concentration (*P* > 0.05), but the 200 μg/mL concentration increased cytotoxicity significantly (*P* < 0.001). By 24 h, highly significant reductions in cytotoxicity were observed with 50 and 200 μg/mL of CIP, LVX, PMB, GEN, OFX and CXM (*P* < 0.0001). CHL used at a concentration of 50 μg/mL had no significant effect on LDH release at either 9 or 24 h (*P* > 0.05), but percentage cytotoxicity was reduced with the higher concentration tested (*P* < 0.05) (**Figure 2**).

At a MOI = 100, there was a significant reduction by 9 h in percentage cytotoxicity with concentrations of 50 and 200 μg/mL of CIP and LVX (*P* < 0.0001) and GEN and OFX (*P* < 0.01) and with CHL (*P* < 0.01, 200 μg/mL concentration only). By contrast, PMB used at 200 μg/mL significantly increased cytotoxicity (*P* < 0.05). By 24 h, a 50 μg/mL concentration of CIP, LVX, OFX, PMB, GEN and OFX significantly reduced cytotoxicity (*P* < 0.0001), but both CXM and CHL had no significant effect (*P* > 0.05). With the higher concentration of 200 μg/mL, significant reductions were observed for all antibiotic treatments (CIP, LVX, GEN *P* < 0.0001; PMB, OFX, CXM and CHL *P* < 0.05).

### hCF Intracellular *P. aeruginosa* Survival during Different Antibiotic Treatment

**FIGURE 3:**
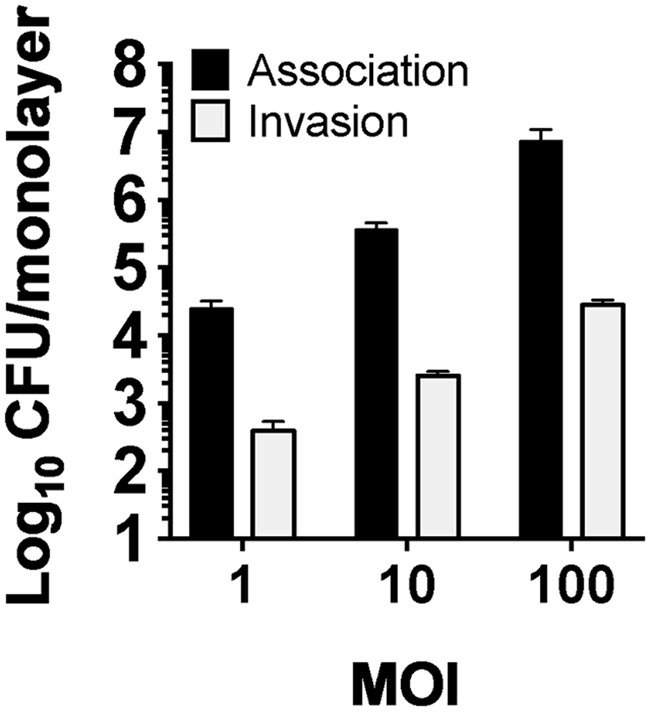
Association of PAO1 to hCF monolayers and subsequent invasion. The graph shows the association of PAO1 to hCF-monolayers after 3 h of infection at MOI = 1, 10, 100, and the levels of invasion measured by quantifying intracellular PAO1 CFU after 90 min of GEN treatment at 200 μg/mL (using a standard gentamicin protection assay). The columns represent the mean CFU/monolayer and the error bars the standard error of the mean (SEM) of *n* = 3 independent experiments.

Recently, we have shown with adherence and gentamicin exclusion assays that *P. aeruginosa* can adhere to primary hCFs and subsequently invade (Cendra et al., 2017). In the current study, MOI-dependent levels of PAO1 adherence and subsequent invasion were observed after 3 h of infection (**Figure 3**). The numbers of invasive PAO1 were quantified as ∼5 × 10^2^ CFU/monolayer with an initial MOI = 1, ∼10^3^ CFU/monolayer for an initial MOI = 10 and ∼5 × 10^4^ CFU/monolayer for an initial MOI = 100 (**Figure 3**).

**FIGURE 4:**
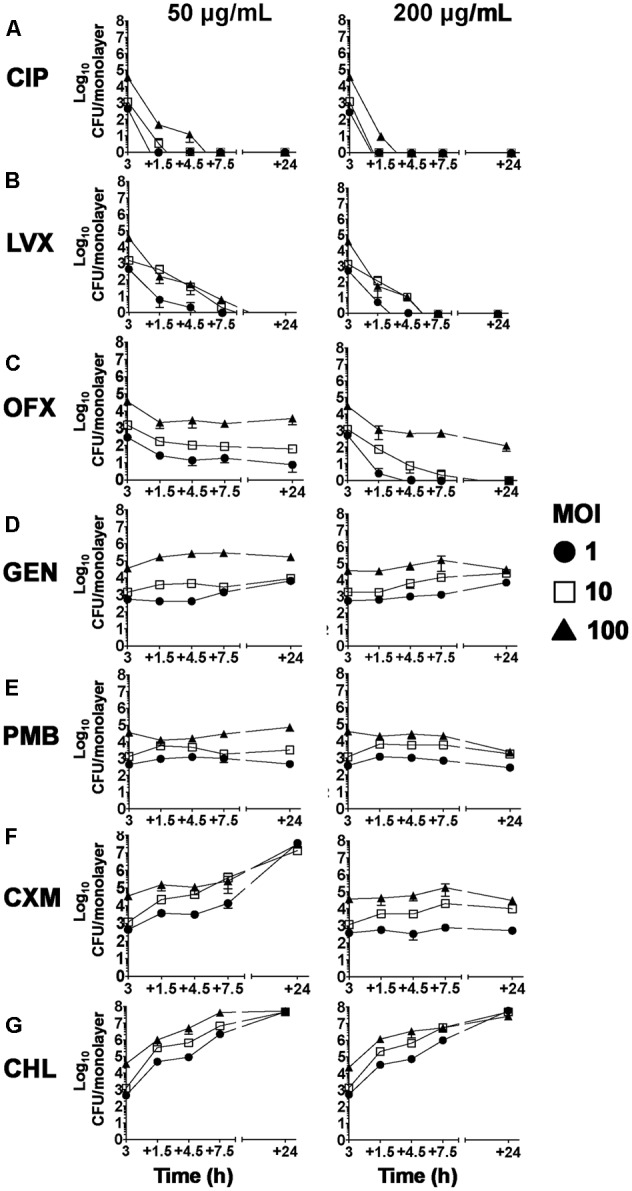
Effect of antibiotic treatments against hCF-intracellular *P. aeruginosa*. Antibiotic treatment at 50 and 200 μg/mL in hCF monolayers infected with PAO1 wild-type (MOI = 1, 10, and 00). The symbols represent the mean and the error bars the SEM from *n* = 3 independent experiments, of intracellular PAO1 CFU that was enumerated by viable counting onto agar plates after 1.5, 4.5, 7.5, and 24 h of each antimicrobial treatment that was added 3 h after initial infection. With CXM and CHL treatment, the PAO1 CFU data represent the sum of associated and invaded bacteria. A one-way ANOVA with Dunnett’s multiple comparison test was used to compare the intracellular CFU numbers at each time point after antibiotic addition (50 and 200 μg/mL) with those recorded at 3 h before addition of antibiotics and for each MOI used. All calculated *P*-values are shown in Supplementary Table S2.

We next tested the hypothesis that the ability of PAO1 to invade hCFs enabled the organism to evade antibiotic chemotherapy, thereby creating a potential reservoir for reactivation of bacterial infection. In order to examine the effects of antibiotics on intracellular bacteria, hCF monolayers were infected with PAO1 (MOI = 1, 10, 100) for 3 h to allow bacterial invasion to occur (**Figure 3**), after which antibiotics (50 or 200 μg/mL) were added to the cultures. The numbers of intracellular PAO1 were then quantified after 1.5, 4.5, 7.5, and 24 h of antibiotic treatment. A clear rank order for antibiotic efficacy was observed against intracellular PAO1 *in vitro*. The fluoroquinolones CIP, LVX and OFX demonstrated the highest antimicrobial activity (**Figure 4A**). Treatment with CIP and LVX (50 and 200 μg/mL) killed >99.99% of all intracellular bacteria by 3 h, regardless of MOI used to infect the monolayers (**Figures 4A,B**). Treatment with OFX (50 or 200 μg/mL) eradicated >99.99% of intracellular bacteria by 3 h from hCF monolayers infected initially with an MOI = 1 (**Figure 4C**). With increasing MOI = 10–100, between ∼10^2^ – ∼10^3^ intracellular bacteria were recovered by 24 h after OFX (50 or 200 μg/mL) treatment, which still represented high antimicrobial activity of >99.9% reduction in CFU (**Figure 4C**).

By contrast to the fluoroquinolones, treatment with GEN, PMB, CXM or CHL failed to clear intracellular PAO1 over the 24 h time course of the experiments. With cells infected with MOI = 1, 10, or 100, ∼10^3^ – ∼10^5^ intracellular bacteria were recovered after treatment for 3 h with GEN or PMB (50 and 200 μg/mL), and these intracellular PAO1 numbers essentially remained unchanged by 24 h (**Figures 4D,E**). CXM treatment at 50 μg/mL could not clear the infection (intracellular or extracellular), and exponential increases in bacterial CFU were observed (**Figure 4F**), and the numbers of intracellular PAO1 enumerated at 3 h (∼10^3^ – ∼10^5^ CFU) in the presence of 200 μg/mL of CXM remained essentially unchanged by 24 h (**Figure 4F**). These experiments confirmed also that PAO1 was resistant to CHL, with exponentially increasing numbers of bacteria recovered over time intracellular and extracellularly (**Figures 2, 4F** and Supplementary Table S2), with cell death occurring early by 9 h.

**FIGURE 5:**
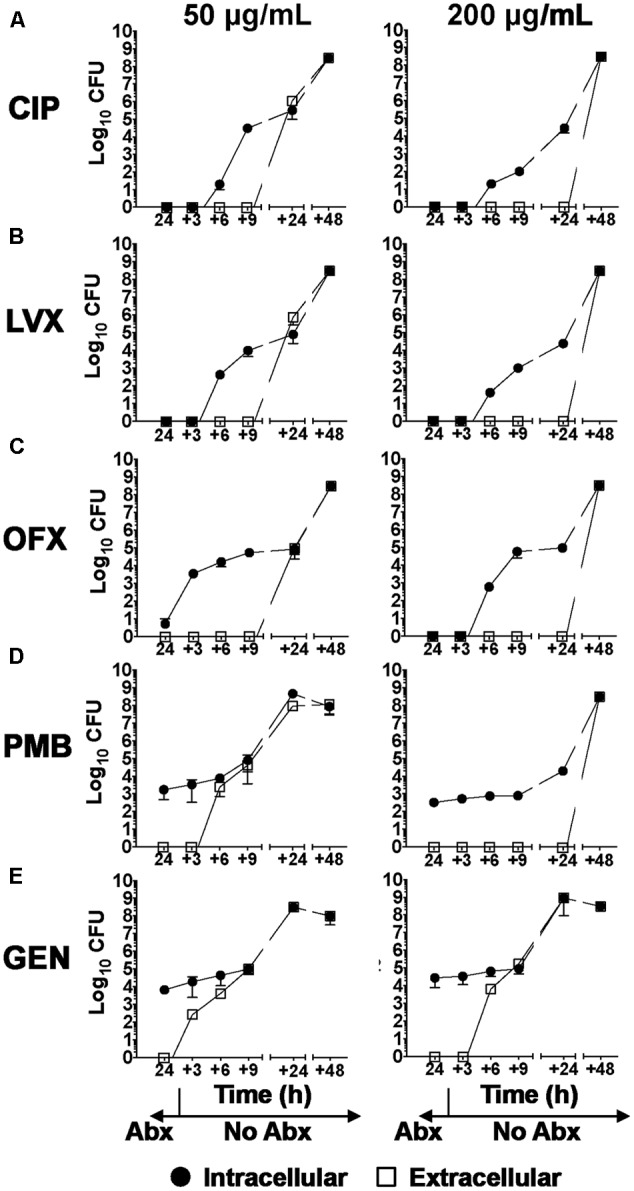
Intracellular and extracellular *P. aeruginosa* growth after cessation of antibiotic treatment of infected hCFs. Intracellular and extracellular PAO1 growth after 3, 6, 9, 24, and 48 h of hCFs infected with PAO1 at MOI = 1 post 24 h treatment with CIP, LVX, PMB, GEN and OFX antibiotics (Abx). CFU enumerated intracellularly by viable counting are shown per monolayer, and measurements of extracellular PAO1 are shown per mL. The symbols represent the mean of extracellular and intracellular PAO1 enumerated in the different treatments and time-points and the error bars the standard error of the mean from *n* = 3 independent experiments. A one way ANOVA with Dunnett’s multiple comparison test was used to compare the intracellular and extracellular CFU numbers at each time point (+3 to +48 h) after removal at 24 h of each antibiotic concentration (50 and 200 μg/mL). All calculated *P* values are shown in Supplementary Table S3.

Clinically, a high incidence of recurrent BK has been observed in patients after cessation of antibiotic treatment (Kaye et al., 2013). We next tested the hypothesis that culture sterility and monolayer integrity were unaffected after removal of the antibiotics at 24 h. hCFs were infected with PAO1 at a MOI = 1 in order to detect logarithmic changes in PAO1 growth, for 3 h prior to the addition of antibiotics, as described above. At 24 h, antibiotic-containing media were removed, the cell monolayers washed and fresh antibiotic-free medium added. Extracellular and intracellular PAO1 growth was then measured at time intervals up to 48 h after removal of antibiotics. We compared the fluoroquinolones CIP, LEV and OFX along with GEN and PMB, but excluded the CXM and CHL antibiotic treatments due to their inability to limit infection. Intracellular PAO1 growth was eventually detected in all monolayers after removal of antibiotics at 24 h. With CIP and LVX-treated monolayers, intracellular PAO1 growth was detected as early as 3 h after antibiotic removal and increased exponentially over time to 24 h (**Figures 5A,B**). With OFX, intracellular PAO1 were recovered from infected hCF-monolayers at 24 h (**Figure 4**) and after antibiotic removal, intracellular CFU numbers showed similar exponential increases up to 24 h (**Figure 5C**). With these three treatments, extracellular bacteria were recovered by 9 h onward after antibiotic removal and CFU numbers increased exponentially (**Figures 5A–C**). For both PMB and GEN, which failed to clear intracellular bacteria after 24 h (**Figure 4**), removal of the antibiotics resulted in increased CFU numbers intracellularly and early recovery of extracellular bacteria (**Figures 5D,E**). Regardless, after removal at 24 h of all the different antibiotics, by 24–48 h later, all the monolayers were destroyed as a consequence of uncontrolled bacterial growth. All the bacteria recovered at 48 and 96 h now represented growth in host cell-free cultures.

### Effect of Antibiotic Treatment on IL-1β Cytokine Release by *P. aeruginosa* -Infected hCFs

**FIGURE 6:**
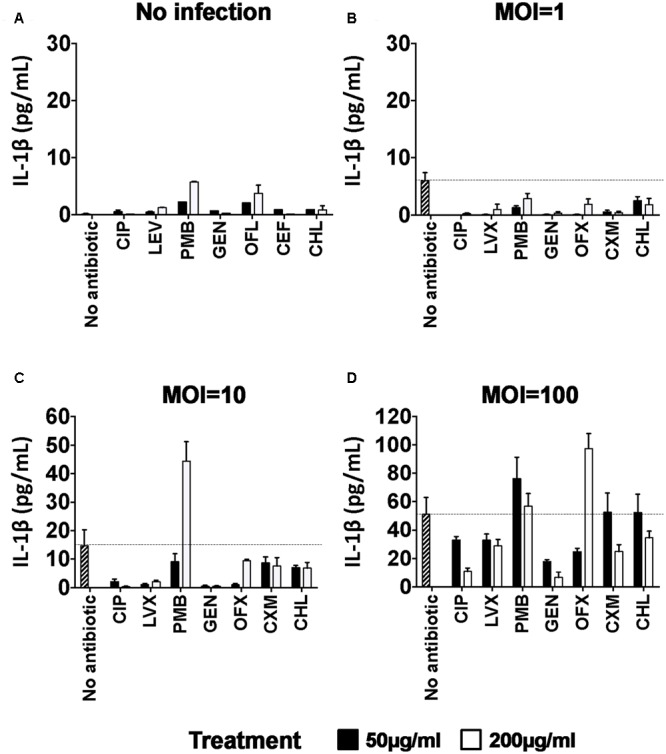
Pro-inflammatory IL-1β cytokine release in PAO1-infected hCFs after antibiotic treatment. MSD-ELISA quantifications of extracellular matured IL-1β in hCFs-supernatants after 9 h of No antibiotic, CIP, LVX, OFX, CXM, GEN, PMB, CHL treatment at 50 and 200 μg/mL of uninfected **(A)** and PAO1-infected monolayers at MOI = 1 **(B)**, = 10 **(C)** and = 100 **(D)** for 3 h. Maximal IL-1β release by hCF infected with PAO1 without antibiotic treatment is marked with a line as reference. The columns represent the mean of extracellular quantification of IL-1β release and the error bars the standard error of the means (SEM) of *n* = 3 independent experiments. A one way ANOVA with Dunnett’s multiple comparison test was used to compare the levels of IL-1β cytokine production in the presence of antibiotic against no antibiotic, for each of the MOI conditions. All calculated *P* values are shown in Supplementary Table S4.

Infection of hCFs with *P. aeruginosa* PAO1 induces significant extracellular release of the cytokine IL-1β (Wong et al., 2011; Cendra et al., 2017). Since exacerbation of the inflammatory response is a critical event during eye infection, we tested the hypothesis that antibiotic treatments not only reduced bacterial infection but were also anti-inflammatory, by influencing IL-1β cytokine production. Our cytotoxicity data demonstrated that after 9 h, untreated PAO1 infection appeared to compromise hCF monolayer integrity; therefore, IL-1β cytokine was measured at this time-point in supernatants of PAO1-infected (MOI = 1, 10, 100) and un-infected hCFs (MOI = 0) monolayers following addition of the different antibiotics after 3 h of infection (**Figure 6**). Baseline levels of IL-1β in uninfected monolayers without antibiotic treatments were ≤0.25 pg/mL (**Figure 6A**). Addition of some of the antibiotics, i.e., PMB, OFX, CXM and CHL, did statistically increase IL-1β release from uninfected monolayers, but the levels never exceeded 6 pg/mL (**Figure 6A** and Supplementary Table S4).

Increased levels of IL-1β production were observed with increasing PAO1 MOI: with MOI = 1, IL-1β levels with no antibiotic were ∼6 pg/mL and addition of all the antibiotics at both concentrations significantly reduced these low levels to ≤3 pg/mL (**Figure 6B** and Supplementary Table S4). With MOI = 10, IL-1β levels with no antibiotic were ∼15–20 pg/mL and addition of all the antibiotics at a concentration of 50 μg/mL either reduced (CIP, LVX, GEN, OFX) or had no significant stimulatory effect (PMB, CXM, CHL) on IL-1β secretion (**Figure 6C** and Supplementary Table S4). Similarly, addition of 200 μg/mL of the antibiotics either reduced (CIP, PMB, GEN) or had no significant stimulatory effect (LVX, OFX, CXM, CHL) on IL-1β levels, except for PMB, which significantly increased (*P* < 0.0001) IL-1β levels (≥40 pg/mL) in the presence of bacteria (**Figure 6C**). With MOI = 100, ∼50–60 pg/mL IL-1β was induced (**Figure 6D**) and none of the antibiotics used at a concentration of 50 μg/mL statistically reduced IL-1β release (Supplementary Table S4). Only CIP and GEN treatment at 200 μg/mL significantly decreased IL-1β release by ≥50% (Supplementary Table S4). PMB, CXM and CHL had no anti-inflammatory effect with similar levels of IL-1β release (*P* > 0.05) measured, whereas addition of 200 μg/mL of OFX was pro-inflammatory, significantly (*P* < 0.001) increasing IL-1β release by ∼2-fold (>100 pg/mL) compared to no antibiotic, infected hCF cells (**Figure 6D** and Supplementary Table S4). Notably, cells infected with MOI = 100 and treated with OFX (200 μg/mL) were morphologically apoptotic, which was not observed in PAO1 infected cells treated with any of the other antibiotics.

## Discussion

The key findings from the current study were that some bactericidal antibiotics routinely used to treat BK failed to eradicate *P*. *aeruginosa* infection of primary hCFs and some exacerbated the host cell cytokine inflammatory response. Bactericidal efficacy of the antibiotics was influenced by the cellular location of the organism. In clinical practice, antibiotic dosing regimens vary significantly from practice to practice, and typically in severe infections, topical antibiotic(s) are administrated hourly to the eye. However, these agents are diluted by the tears with the majority draining through the nasolacrimal duct (O’Brien, 2003). It is not known how much antibiotic penetrates into the infected tissue; in our *in vitro* study, we mimicked a high concentration of antibiotics commonly used to treat BK in the United Kingdom, with a caveat that maintaining high concentrations of antibiotics in essentially closed systems for long periods of time potentially over-estimates antibiotic exposure.

In the current study, we demonstrated that *P. aeruginosa* adhered to primary hCFs *in vitro* and subsequently invaded and replicated intracellularly. Such observations have been reported also with corneal epithelial cells (Fleiszig et al., 1995) and are common with other intracellular pathogens and host cell types (Gresham et al., 2000; Hammerschlag, 2002; Young et al., 2002; Cosma et al., 2003; Flannagan et al., 2016). It is possible that internalization of *P*. *aeruginosa* within corneal epithelial cells and stromal hCFs is a general mechanism employed by this organism to evade immune detection in the cornea and also indirectly to avoid antimicrobial chemotherapy, especially from antibiotics that slowly penetrate eukaryotic cells. The common features of phagocytosis and the ability of some bacteria to survive and replicate intracellularly favors pathogen mobilization within the host and suggests that prolonged antibiotic treatment is likely to be required for complete clearance of infection (Wilson et al., 2002; Cosma et al., 2003). However, *P. aeruginosa*, in common with many other bacterial pathogens, is likely to use other strategies to avoid the bactericidal effects of antimicrobial compounds. These include biofilm-formation, especially on epithelial surfaces alone or in collusion with other opportunistic pathogens such as *Staphylococcus aureus*, the development of genetic resistance, and mechanisms to limit the entry of antimicrobials and/or increase their export through efflux pumps and/or enzymatically modify or destroy their antimicrobial properties (Nikaido, 2009; Blair et al., 2015; Vogwill et al., 2016). Studies have suggested that possible changes in bacterial metabolism that lead to reduced or no replication, may contribute to pathogen defense against antibiotic chemotherapy (Grant and Hung, 2013).

In addition to bacterial resistance strategies, it is possible that differences in the activities of antibiotics against intracellular bacteria may be due to relative accumulated concentrations within the cells (Van Bambeke et al., 2006; Poole, 2011).

In the current study, the three fluoroquinolone antibiotics CIP, LVX and OFX, were the most effective against extracellular and intracellular *P*. *aeruginosa* infection of hCFs, with CIP in particular showing the highest bactericidal activity. Killing of extracellular and intracellular *P. aeruginosa* by fluoroquinolone treatment has been observed also in primary human monocytes (Smith et al., 2000). The intracellular effects of different antibiotics have been tested also in *Pseudomonas* infected THP-1 cells: in this *in vitro* cell model, intracellular activity of antibiotics was dependent not only on the intrinsic growth inhibitory concentrations, but also on the maximal killing capacity of the antibiotic in a specific environment (Buyck et al., 2013). Unlike the fluoroquinolone antibiotics, the aminoglycoside GEN was able to kill extracellular but not intracellular *P*. *aeruginosa*; this may be due to the fact that aminoglycosides are poorly permeable, thus accumulating slowly within cells and only reaching active concentrations with long exposure times (Van Bambeke et al., 2006). Differences in Minimum Inhibitory Concentration (MIC) values for aminoglycosides have been recorded also during *P. aeruginosa* infection of THP-1 cells and were dependent on the cellular localization of the bacterium, with higher concentrations of antibiotics required to kill intracellularly localized organisms (Buyck et al., 2013). PMB could not sterilize *P*. *aeruginosa*-infected hCF monolayers completely: interestingly, this class of antibiotics has been reported to show efficacy against multidrug-resistant *P*. *aeruginosa* (MDR-P) (Poole, 2011), but has a high incidence of side effects, especially contact dermatitis, which limits its use in intensive antibiotic eye drop therapy (Jiaravuthisan and DeKoven, 2008; Poole, 2011). CXM treatment showed high antimicrobial activity against extracellular *P. aeruginosa* growth, but failed to clear intracellular infection. Limited intracellular penetration and accumulation within human cells has been described for this antibiotic, with intracellular CXM concentrations measured ∼100 times lower than the concentration initially administrated (Darouiche and Hamill, 1994). In our model, treatment with the cell-permeant antibiotic CHL was totally ineffective in clearing both intracellular and extracellular bacteria. CHL is bacteriostatic for *P. aeruginosa* and would suppress bacterial replication if present in concentrations higher than the MIC. Since no inhibition of PAO1 growth was observed with CHL treatment, even at a concentration of 200 μg/ml, it is likely that an effective MIC was not reached. In a retrospective review of therapeutic keratoplasty in Singapore, *P. aeruginosa* accounted for 58.7% of refractory microbial keratitis requiring penetrating keratoplasty. Interestingly, all patients were treated initially with gentamicin and a cephalosporin (Ti et al., 2007; Kaye et al., 2013).

In our hCF *in vitro* infection model, we observed eventual intracellular growth of *P. aeruginosa* after cessation of antibiotic treatment, followed by pathogen release and cell death. It has been reported that high levels of recurrent BK follow the cessation of antibiotic treatment (Kaye et al., 2013). This could be due to several factors other than antimicrobial resistance mechanisms, e.g., the early cessation of antibiotic treatment on apparent clearance of clinical infection and sub-optimal levels of accumulated antibiotics within cells. Thus, bacteria residing within both corneal epithelial and fibroblast cells could be the triggers for observed recalcitrant infections. Relapse of *Pseudomonas* keratitis, in particular, occurs in human keratitis after apparent adequate antimicrobial chemotherapy (Van Horn et al., 1978) and the most recent data show that recurrent *P. aeruginosa* spp. are frequently isolated from repeat keratoplasties (Kaye et al., 2013). This indicates that *P. aeruginosa* spp. retention in corneal tissue is a relevant clinical problem. The pathogenesis of *P. aeruginosa* infection of corneal epithelial cells and stromal fibroblast cells share many similarities. Animal models have shown that retention of *P. aeruginosa* spp. in BK occurs within corneal epithelial cells (Lee et al., 2003a) and preliminary *in vitro* studies using electron microscopy, supports the view that a similar process can occur in corneal keratocytes (Elsahn et al., 2013). However, further animal and ideally human studies are required to assess whether this is the case *in vivo*.

A key consideration for antibiotic usage during BK is whether chemotherapy impacts on the inflammatory response induced by *P*. *aeruginosa* infection. A Cochrane review of studies of steroid use in microbial keratitis proposed the earlier use of anti-inflammatories to reduce damaging effects from the infective process (Herretes et al., 2014). Recently, we described the mechanisms whereby *P. aeruginosa* infection induces inflammasome-associated molecules in primary hCFs *in vitro*, which leads principally to the extracellular release of the cytokine IL-1β (Cendra et al., 2017). In our current study, the most effective anti-inflammatory antibiotics were CIP and GEN, which reduced IL-1β production from hCFs infected with high bacterial MOI (=100). All the other antibiotics displayed some anti-inflammatory or non-stimulatory effects, but only with cells infected with lower MOI (≤10). However, the increased levels of extracellular IL-1β quantified from infected monolayers treated with PMB and OFX suggests that these antibiotics are pro-inflammatory in our model. The variability in the hCF inflammatory response to some of the administered antibiotics during active infection *in vitro* is a novel finding and appears to be independent of compound activity against both extracellular and intracellular *P. aeruginosa*. The reported direct activation of the NLRP3 inflammasome-associated molecule by PMB could explain the increased IL-1β release from hCFs during treatment (Allam et al., 2011). The same hypothesis could extend to OFX treatment, and the possibility that both PMB and OFX treatment synergizes with bacterial infection to augment IL-1β production should not be excluded. However, whether these effects involve modulation of the activation of inflammasome-associated molecules is not known. The outcome of increased IL-1β in the presence of some of these antibiotics could be to enhance neutrophil influx (Smith et al., 1997), which could be viewed both as beneficial to the host for pathogen clearance, or potentially damaging as a consequence of eye tissue damage induced by the neutrophils.

Our study also highlighted that antibiotics varied in their cytotoxicity to primary hCF, which was more pronounced for OFX and PMB treatments. The pro-apoptotic effect of OFX could be due to stimulation of intracellular reactive oxygen species (ROS) production (Circu and Aw, 2010), which increases in a concentration- and time-dependent manner after OFX exposure, as observed in other cell-types such as chondrocytes (Sheng et al., 2013). Furthermore, the preservative benkalkonium chloride that was present in the OFX formulation used in this study, but absent in the fluoroquinolone CIP and LVX formulations, has been suggested to potentiate the OFX effect on ROS production and to activate the apoptotic receptor P2X7 (Dutot et al., 2006). In general, the combination of antibiotic and *P. aeruginosa* infection was not synergistic for cell cytotoxicity, and our study demonstrated that several of the antibiotics were able to reduce cytotoxicity, with CIP and LVX particularly effective treatments against high concentrations of bacterial infection.

In summary, our study identified the fluoroquinolone CIP, followed by LVX, as the most effective antibiotics for treating *in vitro Pseudomonas* infection of a monoculture model of primary hCFs. Both antibiotics eliminated extracellular and intracellular bacteria and reduced a cardinal inflammatory cytokine signal, which is observed in BK. The study also suggested that antibiotic concentration regimens must be sufficiently prolonged to eliminate *P. aeruginosa* intracellular infection, in order to minimize the risk of emergent bacteria that could initiate new cycles of infection.

## Author Contributions

MdMC, PH, and MC designed the experimental research. MdMC performed the experiments. MdMC, PH, and MC interpreted and analyzed the data. MC, MdMC, and PH wrote the manuscript. MC and PH contributed equally as PIs to this study.

## Conflict of Interest Statement

The authors declare that the research was conducted in the absence of any commercial or financial relationships that could be construed as a potential conflict of interest.

## Supplementary Material

FIGURE S1 PAO1 susceptibility to antibiotics during planktonic growth in LB medium. An initial PAO1 inoculum of ∼10^5^ CFU/mL, ∼10^6^ CFU/mL and ∼10^7^ CFU/mL was added to LB medium in presence of **(A–G)** CIP, LEV, PMB, GEN, OFX, CXM and CHL at 200, 100, 50, 10, 1, 0.1, and 0.01 μg/mL and optical density (OD) of the bacterial culture measured at λ_600_nm at 0, 1, 2, 3, 4, 5, 6, 7, 8, 9, and 24 h time-points. The symbols represent the mean and the error bars the standard error of the mean (SEM) from *n* = 3 independent experiments.

FIGURE S2 Planktonic growth of PAO1 in LB medium. The graph shows the OD at λ_600_nm of PAO1 from an initial inoculum of ∼10^5^ CFU/mL, ∼10^6^ CFU/mL and ∼10^7^ CFU/mL over-time. The symbols represent the mean and the error bars the standard error of the mean (SEM) from *n* = 3 independent experiments.

FIGURE S3 Extracellular growth of PAO1 during hCF-infection. The graph shows the CFU/mL enumerated extracellularly in infected monolayers at MOI = 1 (∼10^5^ CFU/mL), MOI = 10 (∼10^6^ CFU/mL) and MOI = 100 (∼10^7^ CFU/mL) over-time. The symbols represent the mean and the error bars the SEM from *n* = 3 independent experiments.

FIGURE S4 hCF damage promoted by PAO1 infection and the antibiotics alone. hCF monolayers were infected with PAO1 at MOI = 1, 10, and 100 **(A)** and treated with GEN, CXM, OFX, PMB, CIP, CHL and LVX at 50 and 200 μg/mL **(B)**. LDH release was measured from supernatants after 0, 1, 3, 6, 9, and 24 h of incubation of each condition and the percentage of cytotoxicity was calculated following the manufacturer’s instructions. Non- infected and non-antibiotic treated monolayers were used as controls. The columns represent the mean percentage of cytotoxicity measured as LDH release from monolayers from *n* = 3 independent experiments and the error bars represent the standard error of the means (SEM).
