# Supplementary material for: Effect of Different Antibiotic Chemotherapies on Pseudomonas aeruginosa Infection In Vitro of Primary Human Corneal Fibroblast Cells
Source: Front Microbiol. 2017 Aug 22;8:1614. doi: 10.3389/fmicb.2017.01614 (PMC5572282; doi:10.3389/fmicb.2017.01614)
Supplement: FIGURE S1 — PAO1 susceptibility to antibiotics during planktonic growth in LB medium. An initial PAO1 inoculum of ∼105 CFU/mL, ∼106 CFU/mL and ∼107 CFU/mL was added to LB medium in presence of (A–G) CIP, LEV, PMB, GEN, OFX, CXM and CHL at 200, 100, 50, 10, 1, 0.1, and 0.01 μg/mL and optical density (OD) of the bacterial culture measured at λ600nm at 0, 1, 2, 3, 4, 5, 6, 7, 8, 9, and 24 h time-points. The symbols represent the mean and the error bars the standard error of the mean (SEM) from n = 3 independent experiments. [file Presentation_1.PPTX]

## Slide 1
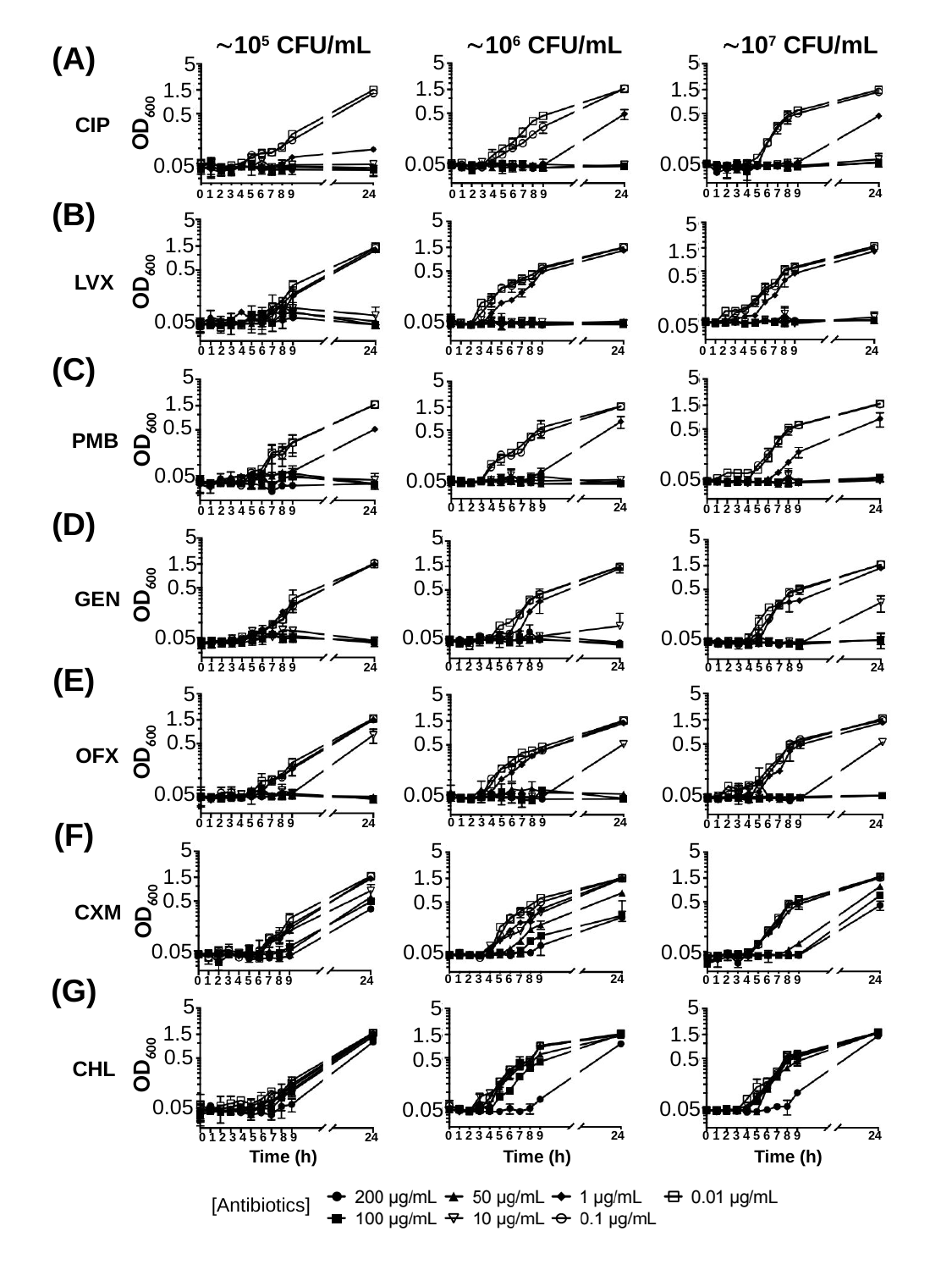

105 CFU/mL
106 CFU/mL
107 CFU/mL
(A)
5
1.5
0.5
0.05
0 1 2 3 4 5 6 7 8 9 24
5
1.5
0.5
0.05
0 1 2 3 4 5 6 7 8 9 24
5
1.5
0.5
OD600
0.05
0 1 2 3 4 5 6 7 8 9 24
CIP
(B)
5
1.5
0.5
OD600
0.05
0 1 2 3 4 5 6 7 8 9 24
5
1.5
0.5
0.05
0 1 2 3 4 5 6 7 8 9 24
5
1.5
0.5
0.05
0 1 2 3 4 5 6 7 8 9 24
LVX
(C)
5
1.5
0.5
OD600
0.05
0 1 2 3 4 5 6 7 8 9 24
5
1.5
0.5
0.05
0 1 2 3 4 5 6 7 8 9 24
5
1.5
0.5
0 1 2 3 4 5 6 7 8 9 24
PMB
0.05
(D)
5
1.5
0.5
OD600
0.05
0 1 2 3 4 5 6 7 8 9 24
5
1.5
0.5
0.05
0 1 2 3 4 5 6 7 8 9 24
5
1.5
0.5
0.05
0 1 2 3 4 5 6 7 8 9 24
GEN
(E)
5
1.5
0.5
0.05
0 1 2 3 4 5 6 7 8 9 24
5
1.5
0.5
OD600
0.05
0 1 2 3 4 5 6 7 8 9 24
5
1.5
0.5
0.05
0 1 2 3 4 5 6 7 8 9 24
OFX
(F)
5
1.5
0.5
0.05
0 1 2 3 4 5 6 7 8 9 24
5
1.5
0.5
0.05
0 1 2 3 4 5 6 7 8 9 24
5
1.5
0.5
0.05
0 1 2 3 4 5 6 7 8 9 24
CXM
OD600
(G)
5
1.5
0.5
OD600
0.05
0 1 2 3 4 5 6 7 8 9 24
5
1.5
0.5
0.05
0 1 2 3 4 5 6 7 8 9 24
5
1.5
0.5
0.05
0 1 2 3 4 5 6 7 8 9 24
CHL
Time (h)
Time (h)
Time (h)
[Antibiotics]
