# Supplementary figures and images for: Effect of Different Antibiotic Chemotherapies on Pseudomonas aeruginosa Infection In Vitro of Primary Human Corneal Fibroblast Cells
Source: Front Microbiol. 2017 Aug 22;8:1614. doi: 10.3389/fmicb.2017.01614 (PMC5572282; doi:10.3389/fmicb.2017.01614)

## Slide 1
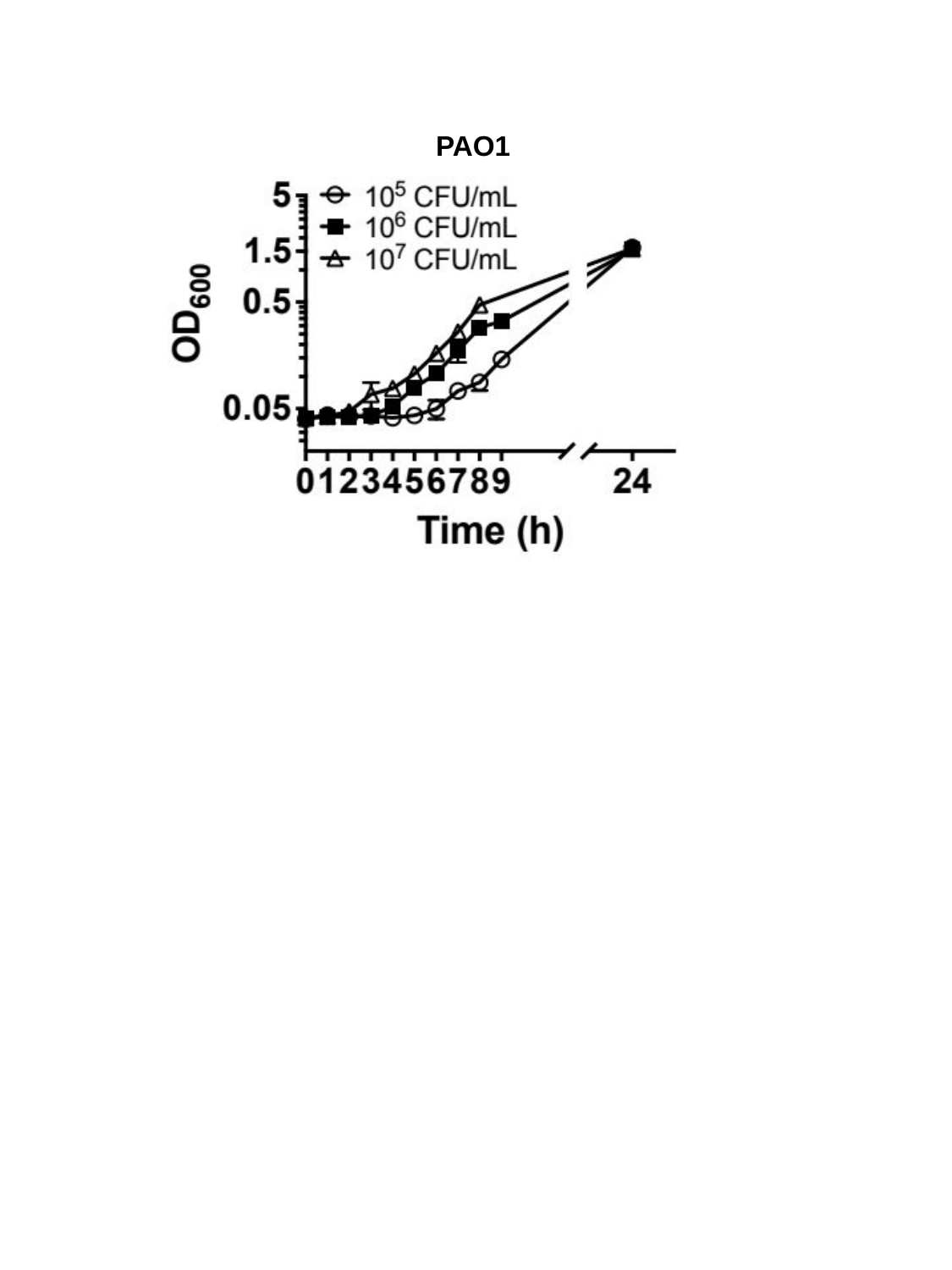

PAO1

Supplement: FIGURE S2 — Planktonic growth of PAO1 in LB medium. The graph shows the OD at λ600nm of PAO1 from an initial inoculum of ∼105 CFU/mL, ∼106 CFU/mL and ∼107 CFU/mL over-time. The symbols represent the mean and the error bars the standard error of the mean (SEM) from n = 3 independent experiments. [file Presentation_2.PPTX]

## Slide 1
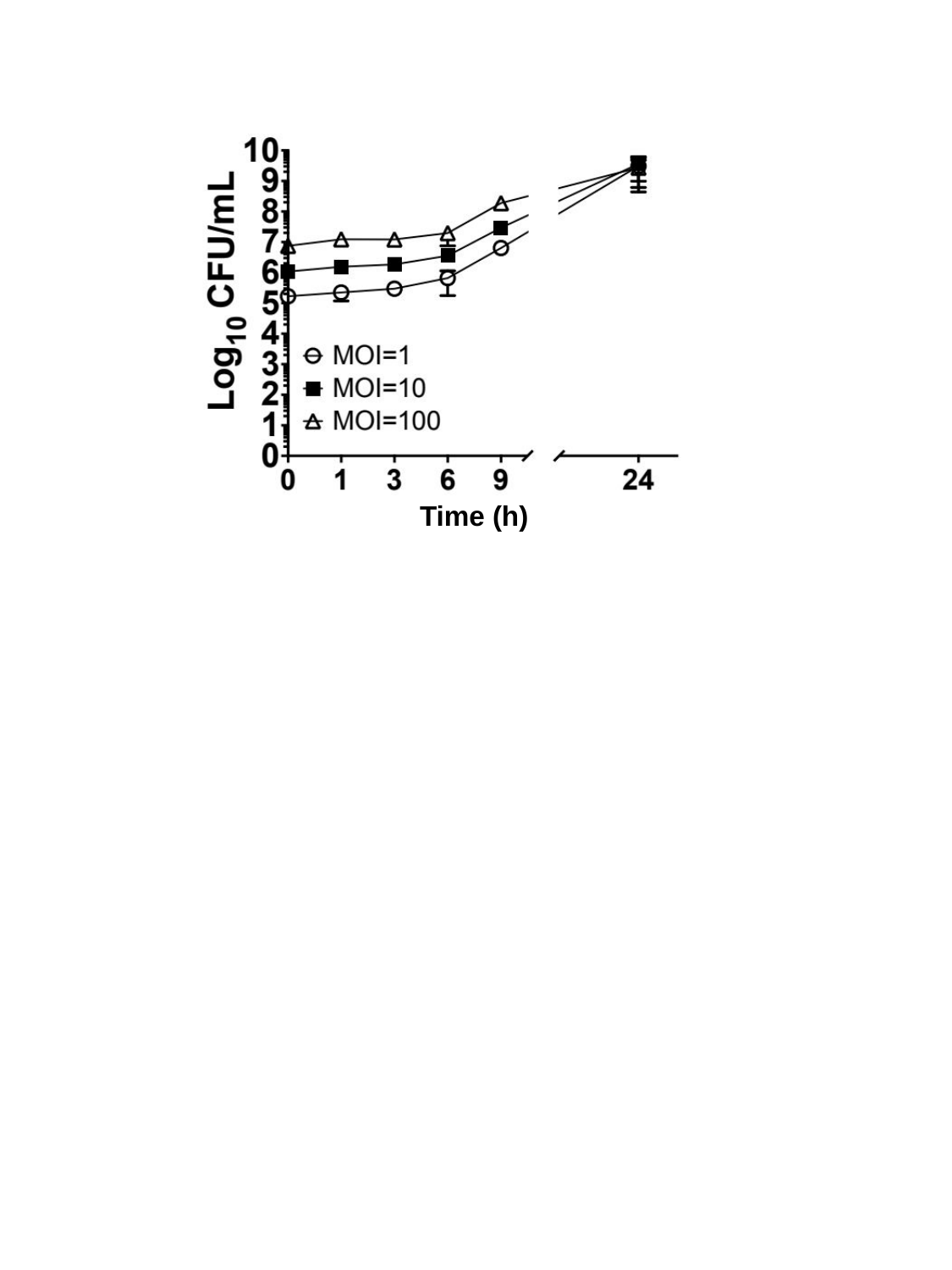

Time (h)

Supplement: FIGURE S3 — Extracellular growth of PAO1 during hCF-infection. The graph shows the CFU/mL enumerated extracellularly in infected monolayers at MOI = 1 (∼105 CFU/mL), MOI = 10 (∼106 CFU/mL) and MOI = 100 (∼107 CFU/mL) over-time. The symbols represent the mean and the error bars the SEM from n = 3 independent experiments. [file Presentation_3.PPTX]

## Slide 1
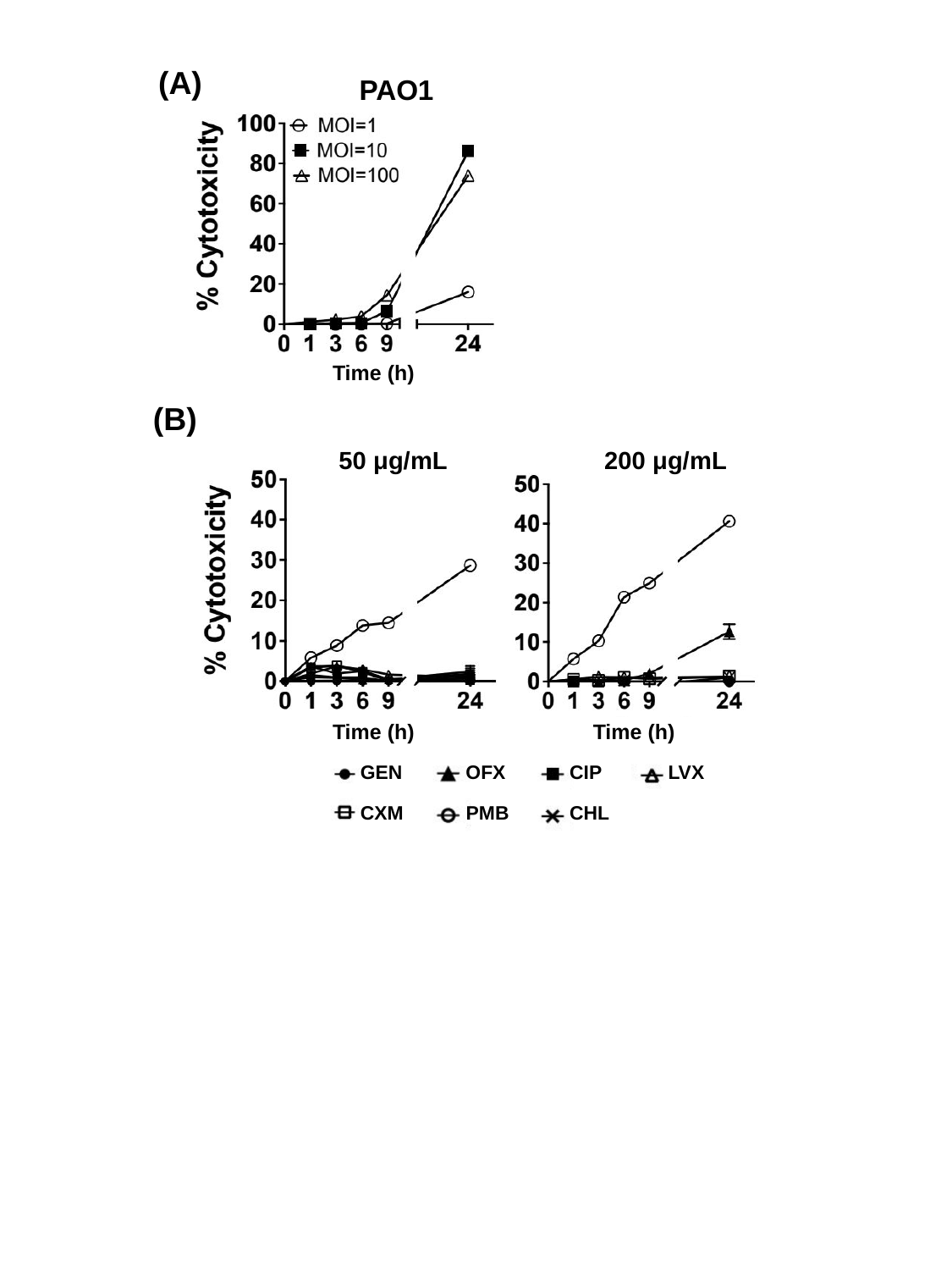

(A)
PAO1
Time (h)
(B)
50 μg/mL
200 μg/mL
Time (h)
Time (h)
GEN
OFX
CIP
LVX
CXM
PMB
CHL

Supplement: FIGURE S4 — hCF damage promoted by PAO1 infection and the antibiotics alone. hCF monolayers were infected with PAO1 at MOI = 1, 10, and 100 (A) and treated with GEN, CXM, OFX, PMB, CIP, CHL and LVX at 50 and 200 μg/mL (B). LDH release was measured from supernatants after 0, 1, 3, 6, 9, and 24 h of incubation of each condition and the percentage of cytotoxicity was calculated following the manufacturer’s instructions. Non- infected and non-antibiotic treated monolayers were used as controls. The columns represent the mean percentage of cytotoxicity measured as LDH release from monolayers from n = 3 independent experiments and the error bars represent the standard error of the means (SEM). [file Presentation_4.PPTX]
