# Supplementary material for: Effect of Different Antibiotic Chemotherapies on Pseudomonas aeruginosa Infection In Vitro of Primary Human Corneal Fibroblast Cells
Source: Front Microbiol. 2017 Aug 22;8:1614. doi: 10.3389/fmicb.2017.01614 (PMC5572282; doi:10.3389/fmicb.2017.01614)
Supplement: Supplementary file 5 [file Table_1.DOCX]

**Supplementary Table 1. PAO1 susceptibility to CIP, LVX, PMB, GEN, OFX, CXM and CHL antibiotics after 24 h of planktonic growth in LB medium.** Tables show the optical density (OD) of the bacterial culture measured at λ = _600_nm after 24 h of growth in LB medium with an initial PAO1 inoculum of ~10^5^ CFU/mL, ~10^6^ CFU/mL and ~10^7^ CFU/mL. The antibiotic concentrations tested were 200, 100, 50, 10, 1, 0.1 and 0.01 μg/mL. The values showed are the mean ± standard error of the mean (SEM) from n=3 independent experiments. OD values for untreated PAO1 cultures at 24h were >1.5 (see Supplementary Figure 2).

| **~10^5^ CFU/mL PAO-1** | | | | | | | |
| --- | --- | --- | --- | --- | --- | --- | --- |
|  | **Antibiotic Concentration** | | | | | | |
| **Antibiotic** | 200 μg/mL | 100 μg/mL | 50 μg/mL | 10 μg/mL | 1 μg/mL | 0.1 μg/mL | 0.01 μg/mL |
| CIP | 0.039 ± 0.007 | 0.041 ± 0.006 | 0.042 ± 0.003 | 0.044 ± 0.006 | 0.086 ± 0.001 | 1.226 ± 0.061 | 1.491 ± 0.071 |
| LVX | 0.040 ± 0.002 | 0.038 ± 0.002 | 0.040 ± 0.005 | 0.039 ± 0.020 | 1.241 ± 0.009 | 1.510 ± 0.149 | 1.631 ± 0.215 |
| PMB | 0.041 ± 0.001 | 0.039 ± 0.003 | 0.041 ± 0.001 | 0.063 ± 0.013 | 0.470 ± 0.045 | 1.560 ± 0.024 | 1.590 ± 0.059 |
| GEN | 0.043 ± 0.001 | 0.043 ± 0.008 | 0.040 ± 0.004 | 0.041 ± 0.001 | 1.674 ± 0.170 | 1.601 ± 0.018 | 1.604 ± 0,093 |
| OFX | 0.040 ± 0.005 | 0.039 ± 0.004 | 0.042 ± 0.001 | 0.854 ± 0.164 | 1.418 ± 0.092 | 1.624 ± 0.054 | 1.621 ± 0.020 |
| CXM | 0.350 ± 0.002 | 0.469 ± 0.017 | 0.571 ± 0.029 | 0.615 ± 0.188 | 1.527 ± 0.127 | 1.571 ± 0.016 | 1.639 ± 0.052 |
| CHL | 1.039 ± 0.008 | 1.366 ± 0.081 | 1.544 ± 0.035 | 1.594 ± 0.037 | 1.646 ± 0.078 | 1.627 ± 0.029 | 1.569 ± 0.029 |

| **~10^6^ CFU/mL PAO-1** | | | | | | | |
| --- | --- | --- | --- | --- | --- | --- | --- |
|  | **Antibiotic Concentration** | | | | | | |
| **Antibiotic** | 200 μg/mL | 100 μg/mL | 50 μg/mL | 10 μg/mL | 1 μg/mL | 0.1 μg/mL | 0.01 μg/mL |
| CIP | 0.040 ± 0.003 | 0.042 ± 0.012 | 0.041 ± 0.018 | 0.044 ± 0.003 | 0.406 ± 0.077 | 1.570 ± 0.015 | 1.640 ± 0.102 |
| LVX | 0.049 ± 0.003 | 0.043 ± 0.001 | 0.056 ± 0.006 | 0.048 ± 0.002 | 1.334 ± 0.039 | 1.553 ± 0.064 | 1.558 ± 0.037 |
| PMB | 0.039 ± 0.001 | 0.038 ± 0.002 | 0.043 ± 0.001 | 0.047 ± 0.002 | 0.605 ± 0.144 | 1.549 ± 0.023 | 1.549 ± 0.033 |
| GEN | 0.043 ± 0.001 | 0.041 ± 0.003 | 0.035 ± 0.003 | 0.044 ± 0.053 | 1.434 ± 0.178 | 1.601 ± 0.046 | 1.600 ± 0.147 |
| OFX | 0.044 ± 0.003 | 0.036 ± 0.003 | 0.042 ± 0.001 | 0.906 ± 0.014 | 1.418 ± 0.105 | 1.622 ± 0.035 | 1.612 ± 0.051 |
| CXM | 0.448 ± 0.198 | 0.502 ± 0.044 | 0.629 ± 0.063 | 0.992 ± 0.048 | 1.527 ± 0.089 | 1.571 ± 0.052 | 1.639 ± 0.072 |
| CHL | 1.055 ± 0.064 | 1.368 ± 0.029 | 1.545 ± 0.088 | 1.594 ± 0.082 | 1.646 ± 0.059 | 1.627 ± 0.052 | 1.628 ± 0.046 |

| **~10^7^ CFU/mL PAO-1** | |
| --- | --- |
|  | **Antibiotic Concentration** |

| **Antibiotic** | 200 μg/mL | 100 μg/mL | 50 μg/mL | 10 μg/mL | 1 μg/mL | 0.1 μg/mL | 0.01 μg/mL |
| --- | --- | --- | --- | --- | --- | --- | --- |
| CIP | 0.047 ± 0.005 | 0.044 ± 0.004 | 0.042 ± 0.003 | 0.042 ± 0.013 | 0.511 ± 0.034 | 1.352 ± 0.081 | 1.586 ± 0.042 |
| LVX | 0.051 ± 0.002 | 0.053 ± 0.003 | 0.052 ± 0.003 | 0.068 ± 0.011 | 1.286 ± 0.085 | 1.651 ± 0.024 | 1.608 ± 0.072 |
| PMB | 0.055 ± 0.006 | 0.057 ± 0.009 | 0.048 ± 0.009 | 0.047 ± 0.001 | 0.982 ± 0.253 | 1.581 ± 0.015 | 1.652 ± 0.050 |
| GEN | 0.042 ± 0.009 | 0.049 ± 0.009 | 0.046 ± 0.005 | 0.205 ± 0.069 | 1.373 ± 0.032 | 1.374 ± 0.009 | 1.584 ± 0.003 |
| OFX | 0.046 ± 0.002 | 0.047 ± 0.001 | 0.049 ± 0.001 | 0.542 ± 0,072 | 1.321 ± 0.097 | 1.475 ± 0.108 | 1.588 ±0.099 |
| CXM | 0.491 ± 0.089 | 0.631 ± 0.064 | 1.083 ± 0.079 | 1.554 ± 0.071 | 1.501 ± 0.077 | 1.512 ± 0.049 | 1.630 ± 0.028 |
| CHL | 1.464 ± 0.056 | 1.594 ± 0.066 | 1.629 ± 0.012 | 1.661 ± 0.027 | 1.654 ± 0.030 | 1.651 ± 0.011 | 1.656 ± 0.034 |
