# Supplementary material for: Effect of Different Antibiotic Chemotherapies on Pseudomonas aeruginosa Infection In Vitro of Primary Human Corneal Fibroblast Cells
Source: Front Microbiol. 2017 Aug 22;8:1614. doi: 10.3389/fmicb.2017.01614 (PMC5572282; doi:10.3389/fmicb.2017.01614)
Supplement: Supplementary file 6 [file Table_2.DOCX]

**Supplementary Table 2. One-way ANOVA with Dunnett’s multiple comparison test for data shown in Figure 4**

| **Antibiotic** | **Concentration** | **MOI** | **Test (3h versus +h)** | **Adjusted P value** | **Significant** | **CFU numbers** |
| --- | --- | --- | --- | --- | --- | --- |
| **Ciprofloxacin** | 50µg/mL | 1 | 3 vs. +1.5 | 0.0016 | Yes | Reduced |
|  |  |  | 3 vs. +4.5 | 0.0016 | Yes | Reduced |
|  |  |  | 3 vs. +7.5 | 0.0016 | Yes | Reduced |
|  |  |  | 3 vs. +24 | 0.0016 | Yes | Reduced |
|  |  |  |  |  |  |  |
|  | 50µg/mL | 10 | 3 vs. +1.5 | 0.0005 | Yes | Reduced |
|  |  |  | 3 vs. +4.5 | 0.0004 | Yes | Reduced |
|  |  |  | 3 vs. +7.5 | 0.0004 | Yes | Reduced |
|  |  |  | 3 vs. +24 | 0.0004 | Yes | Reduced |
|  |  |  |  |  |  |  |
|  | 50µg/mL | 100 | 3 vs. +1.5 | 0.0001 | Yes | Reduced |
|  |  |  | 3 vs. +4.5 | 0.0001 | Yes | Reduced |
|  |  |  | 3 vs. +7.5 | 0.0001 | Yes | Reduced |
|  |  |  | 3 vs. +24 | 0.0001 | Yes | Reduced |
|  |  |  |  |  |  |  |
|  | 200 µg/mL | 1 | 3 vs. +1.5 | 0.0014 | Yes | Reduced |
|  |  |  | 3 vs. +4.5 | 0.0014 | Yes | Reduced |
|  |  |  | 3 vs. +7.5 | 0.0014 | Yes | Reduced |
|  |  |  | 3 vs. +24 | 0.0014 | Yes | Reduced |
|  |  |  |  |  |  |  |
|  | 200 µg/mL | 10 | 3 vs. +1.5 | 0.0001 | Yes | Reduced |
|  |  |  | 3 vs. +4.5 | 0.0001 | Yes | Reduced |
|  |  |  | 3 vs. +7.5 | 0.0001 | Yes | Reduced |
|  |  |  | 3 vs. +24 | 0.0001 | Yes | Reduced |
|  |  |  |  |  |  |  |
|  | 200 µg/mL | 100 | 3 vs. +1.5 | 0.0001 | Yes | Reduced |
|  |  |  | 3 vs. +4.5 | 0.0002 | Yes | Reduced |
|  |  |  | 3 vs. +7.5 | 0.0002 | Yes | Reduced |
|  |  |  | 3 vs. +24 | 0.0002 | Yes | Reduced |
|  |  |  |  |  |  |  |
|  |  |  |  |  |  |  |
|  |  |  |  |  |  |  |
| **Antibiotic** | **Concentration** | **MOI** | **Test (3h versus +h)** | **Adjusted P value** | **Significant** | **CFU numbers** |
| **Levofloxacin** | 50µg/mL | 1 | 3 vs. +1.5 | 0.0007 | Yes | Reduced |
|  |  |  | 3 vs. +4.5 | 0.0007 | Yes | Reduced |
|  |  |  | 3 vs. +7.5 | 0.0011 | Yes | Reduced |
|  |  |  | 3 vs. +24 | 0.0021 | Yes | Reduced |
|  |  |  |  |  |  |  |
|  | 50µg/mL | 10 | 3 vs. +1.5 | 0.0014 | Yes | Reduced |
|  |  |  | 3 vs. +4.5 | 0.0001 | Yes | Reduced |
|  |  |  | 3 vs. +7.5 | 0.0001 | Yes | Reduced |
|  |  |  | 3 vs. +24 | 0.0001 | Yes | Reduced |
|  |  |  |  |  |  |  |
|  | 50µg/mL | 100 | 3 vs. +1.5 | 0.0001 | Yes | Reduced |
|  |  |  | 3 vs. +4.5 | 0.0001 | Yes | Reduced |
|  |  |  | 3 vs. +7.5 | 0.0001 | Yes | Reduced |
|  |  |  | 3 vs. +24 | 0.0001 | Yes | Reduced |
|  |  |  |  |  |  |  |
|  | 200 µg/mL | 1 | 3 vs. +1.5 | 0.0001 | Yes | Reduced |
|  |  |  | 3 vs. +4.5 | 0.0001 | Yes | Reduced |
|  |  |  | 3 vs. +7.5 | 0.0001 | Yes | Reduced |
|  |  |  | 3 vs. +24 | 0.0001 | Yes | Reduced |
|  |  |  |  |  |  |  |
|  | 200 µg/mL | 10 | 3 vs. +1.5 | 0.0001 | Yes | Reduced |
|  |  |  | 3 vs. +4.5 | 0.0001 | Yes | Reduced |
|  |  |  | 3 vs. +7.5 | 0.0001 | Yes | Reduced |
|  |  |  | 3 vs. +24 | 0.0001 | Yes | Reduced |
|  |  |  |  |  |  |  |
|  | 200 µg/mL | 100 | 3 vs. +1.5 | 0.0001 | Yes | Reduced |
|  |  |  | 3 vs. +4.5 | 0.0001 | Yes | Reduced |
|  |  |  | 3 vs. +7.5 | 0.0001 | Yes | Reduced |
|  |  |  | 3 vs. +24 | 0.0001 | Yes | Reduced |
|  |  |  |  |  |  |  |
|  |  |  |  |  |  |  |
|  |  |  |  |  |  |  |
|  |  |  |  |  |  |  |
|  |  |  |  |  |  |  |
| **Antibiotic** | **Concentration** | **MOI** | **Test (3h versus +h)** | **Adjusted P value** | **Significant** | **CFU numbers** |
| **Ofloxacin** | 50µg/mL | 1 | 3 vs. +1.5 | 0.0001 | Yes | Reduced |
|  |  |  | 3 vs. +4.5 | 0.0001 | Yes | Reduced |
|  |  |  | 3 vs. +7.5 | 0.0001 | Yes | Reduced |
|  |  |  | 3 vs. +24 | 0.0001 | Yes | Reduced |
|  |  |  |  |  |  |  |
|  | 50µg/mL | 10 | 3 vs. +1.5 | 0.0061 | Yes | Reduced |
|  |  |  | 3 vs. +4.5 | 0.0085 | Yes | Reduced |
|  |  |  | 3 vs. +7.5 | 0.0078 | Yes | Reduced |
|  |  |  | 3 vs. +24 | 0.0008 | Yes | Reduced |
|  |  |  |  |  |  |  |
|  | 50µg/mL | 100 | 3 vs. +1.5 | 0.0001 | Yes | Reduced |
|  |  |  | 3 vs. +4.5 | 0.0001 | Yes | Reduced |
|  |  |  | 3 vs. +7.5 | 0.0001 | Yes | Reduced |
|  |  |  | 3 vs. +24 | 0.0002 | Yes | Reduced |
|  |  |  |  |  |  |  |
|  | 200 µg/mL | 1 | 3 vs. +1.5 | 0.0001 | Yes | Reduced |
|  |  |  | 3 vs. +4.5 | 0.0001 | Yes | Reduced |
|  |  |  | 3 vs. +7.5 | 0.0001 | Yes | Reduced |
|  |  |  | 3 vs. +24 | 0.0001 | Yes | Reduced |
|  |  |  |  |  |  |  |
|  | 200 µg/mL | 10 | 3 vs. +1.5 | 0.0001 | Yes | Reduced |
|  |  |  | 3 vs. +4.5 | 0.0001 | Yes | Reduced |
|  |  |  | 3 vs. +7.5 | 0.0001 | Yes | Reduced |
|  |  |  | 3 vs. +24 | 0.0001 | Yes | Reduced |
|  |  |  |  |  |  |  |
|  | 200 µg/mL | 100 | 3 vs. +1.5 | 0.0001 | Yes | Reduced |
|  |  |  | 3 vs. +4.5 | 0.0001 | Yes | Reduced |
|  |  |  | 3 vs. +7.5 | 0.0001 | Yes | Reduced |
|  |  |  | 3 vs. +24 | 0.0001 | Yes | Reduced |
|  |  |  |  |  |  |  |
|  |  |  |  |  |  |  |
|  |  |  |  |  |  |  |
|  |  |  |  |  |  |  |
| **Antibiotic** | **Concentration** | **MOI** | **Test (3h versus +h)** | **Adjusted P value** | **Significant** | **CFU numbers** |
| **Gentamicin** | 50µg/mL | 1 | 3 vs. +1.5 | 0.9991 | No | Similar |
|  |  |  | 3 vs. +4.5 | 0.9993 | No | Similar |
|  |  |  | 3 vs. +7.5 | 0.5116 | No | Similar |
|  |  |  | 3 vs. +24 | 0.0001 | Yes | Increased |
|  |  |  |  |  |  |  |
|  | 50µg/mL | 10 | 3 vs. +1.5 | 0.1636 | No | Similar |
|  |  |  | 3 vs. +4.5 | 0.0558 | No | Similar |
|  |  |  | 3 vs. +7.5 | 0.6113 | No | Similar |
|  |  |  | 3 vs. +24 | 0.0001 | Yes | Increased |
|  |  |  |  |  |  |  |
|  | 50µg/mL | 100 | 3 vs. +1.5 | 0.7362 | No | Similar |
|  |  |  | 3 vs. +4.5 | 0.2568 | No | Similar |
|  |  |  | 3 vs. +7.5 | 0.1719 | No | Similar |
|  |  |  | 3 vs. +24 | 0.7410 | No | Similar |
|  |  |  |  |  |  |  |
|  | 200 µg/mL | 1 | 3 vs. +1.5 | 0.9971 | No | Similar |
|  |  |  | 3 vs. +4.5 | 0.4961 | No | Similar |
|  |  |  | 3 vs. +7.5 | 0.1982 | No | Similar |
|  |  |  | 3 vs. +24 | 0.0001 | Yes | Increased |
|  |  |  |  |  |  |  |
|  | 200 µg/mL | 10 | 3 vs. +1.5 | 0.9999 | No | Similar |
|  |  |  | 3 vs. +4.5 | 0.9002 | No | Similar |
|  |  |  | 3 vs. +7.5 | 0.2117 | No | Increased |
|  |  |  | 3 vs. +24 | 0.0051 | Yes | Increased |
|  |  |  |  |  |  |  |
|  | 200 µg/mL | 100 | 3 vs. +1.5 | 0.9999 | No | Similar |
|  |  |  | 3 vs. +4.5 | 0.9536 | No | Similar |
|  |  |  | 3 vs. +7.5 | 0.2780 | No | Similar |
|  |  |  | 3 vs. +24 | 0.9999 | No | Similar |
|  |  |  |  |  |  |  |
|  |  |  |  |  |  |  |
|  |  |  |  |  |  |  |
|  |  |  |  |  |  |  |
| **Antibiotic** | **Concentration** | **MOI** | **Test (3h versus +h)** | **Adjusted P value** | **Significant** | **CFU numbers** |
| **Polymyxin B** | 50µg/mL | 1 | 3 vs. +1.5 | 0.5969 | No | Similar |
|  |  |  | 3 vs. +4.5 | 0.2753 | No | Similar |
|  |  |  | 3 vs. +7.5 | 0.5533 | No | Similar |
|  |  |  | 3 vs. +24 | 0.9999 | No | Similar |
|  |  |  |  |  |  |  |
|  | 50µg/mL | 10 | 3 vs. +1.5 | 0.0001 | Yes | Increased |
|  |  |  | 3 vs. +4.5 | 0.0007 | Yes | Increased |
|  |  |  | 3 vs. +7.5 | 0.8565 | No | Similar |
|  |  |  | 3 vs. +24 | 0.0431 | No | Similar |
|  |  |  |  |  |  |  |
|  | 50µg/mL | 100 | 3 vs. +1.5 | 0.4758 | No | Similar |
|  |  |  | 3 vs. +4.5 | 0.6563 | No | Similar |
|  |  |  | 3 vs. +7.5 | 0.9897 | No | Similar |
|  |  |  | 3 vs. +24 | 0.1651 | No | Similar |
|  |  |  |  |  |  |  |
|  | 200 µg/mL | 1 | 3 vs. +1.5 | 0.0286 | Yes | Increased |
|  |  |  | 3 vs. +4.5 | 0.0976 | No | Similar |
|  |  |  | 3 vs. +7.5 | 0.5393 | No | Similar |
|  |  |  | 3 vs. +24 | 0.9979 | No | Similar |
|  |  |  |  |  |  |  |
|  | 200 µg/mL | 10 | 3 vs. +1.5 | 0.0015 | Yes | Increased |
|  |  |  | 3 vs. +4.5 | 0.0113 | Yes | Increased |
|  |  |  | 3 vs. +7.5 | 0.0087 | Yes | Increased |
|  |  |  | 3 vs. +24 | 0.9949 | No | Similar |
|  |  |  |  |  |  |  |
|  | 200 µg/mL | 100 | 3 vs. +1.5 | 0.4271 | No | Similar |
|  |  |  | 3 vs. +4.5 | 0.8432 | No | Similar |
|  |  |  | 3 vs. +7.5 | 0.5633 | No | Similar |
|  |  |  | 3 vs. +24 | 0.0958 | No | Similar |
|  |  |  |  |  |  |  |
|  |  |  |  |  |  |  |
|  |  |  |  |  |  |  |
|  |  |  |  |  |  |  |
| **Antibiotic** | **Concentration** | **MOI** | **Test (3h versus +h)** | **Adjusted P value** | **Significant** | **CFU numbers** |
| **Cefuroxime** | 50µg/mL | 1 | 3 vs. +1.5 | 0.9999 | No | Increased |
|  |  |  | 3 vs. +4.5 | 0.9999 | No | Increased |
|  |  |  | 3 vs. +7.5 | 0.9999 | No | Increased |
|  |  |  | 3 vs. +24 | 0.0020 | Yes | Increased |
|  |  |  |  |  |  |  |
|  | 50µg/mL | 10 | 3 vs. +1.5 | 0.9999 | No | Increased |
|  |  |  | 3 vs. +4.5 | 0.9999 | No | Increased |
|  |  |  | 3 vs. +7.5 | 0.9870 | No | Increased |
|  |  |  | 3 vs. +24 | 0.0001 | Yes | Increased |
|  |  |  |  |  |  |  |
|  | 50µg/mL | 100 | 3 vs. +1.5 | 0.9999 | No | Similar |
|  |  |  | 3 vs. +4.5 | 0.9999 | No | Similar |
|  |  |  | 3 vs. +7.5 | 0.9999 | No | Similar |
|  |  |  | 3 vs. +24 | 0.0001 | Yes | Increased |
|  |  |  |  |  |  |  |
|  | 200 µg/mL | 1 | 3 vs. +1.5 | 0.8699 | No | Similar |
|  |  |  | 3 vs. +4.5 | 0.9980 | No | Similar |
|  |  |  | 3 vs. +7.5 | 0.3933 | No | Similar |
|  |  |  | 3 vs. +24 | 0.9253 | No | Similar |
|  |  |  |  |  |  |  |
|  | 200 µg/mL | 10 | 3 vs. +1.5 | 0.7193 | No | Similar |
|  |  |  | 3 vs. +4.5 | 0.7541 | No | Similar |
|  |  |  | 3 vs. +7.5 | 0.0004 | Yes | Increased |
|  |  |  | 3 vs. +24 | 0.0884 | No | Similar |
|  |  |  |  |  |  |  |
|  | 200 µg/mL | 100 | 3 vs. +1.5 | 0.9999 | No | Similar |
|  |  |  | 3 vs. +4.5 | 0.9961 | No | Similar |
|  |  |  | 3 vs. +7.5 | 0.2963 | No | Similar |
|  |  |  | 3 vs. +24 | 0.9999 | No | Similar |
|  |  |  |  |  |  |  |
|  |  |  |  |  |  |  |
|  |  |  |  |  |  |  |
|  |  |  |  |  |  |  |
| **Antibiotic** | **Concentration** | **MOI** | **Test (3h versus +h)** | **Adjusted P value** | **Significant** | **CFU numbers** |
| **Chloramphenicol** | 50µg/mL | 1 | 3 vs. +1.5 | 0.9999 | No | Increased |
|  |  |  | 3 vs. +4.5 | 0.9999 | No | Increased |
|  |  |  | 3 vs. +7.5 | 0.9977 | No | Increased |
|  |  |  | 3 vs. +24 | 0.0006 | Yes | Increased |
|  |  |  |  |  |  |  |
|  | 50µg/mL | 10 | 3 vs. +1.5 | 0.9999 | No | Increased |
|  |  |  | 3 vs. +4.5 | 0.9999 | No | Increased |
|  |  |  | 3 vs. +7.5 | 0.8594 | No | Increased |
|  |  |  | 3 vs. +24 | 0.0003 | Yes | Increased |
|  |  |  |  |  |  |  |
|  | 50µg/mL | 100 | 3 vs. +1.5 | 0.9999 | No | Increased |
|  |  |  | 3 vs. +4.5 | 0.9724 | No | Increased |
|  |  |  | 3 vs. +7.5 | 0.0034 | Yes | Increased |
|  |  |  | 3 vs. +24 | 0.0004 | Yes | Increased |
|  |  |  |  |  |  |  |
|  | 200 µg/mL | 1 | 3 vs. +1.5 | 0.9999 | No | Increased |
|  |  |  | 3 vs. +4.5 | 0.9999 | No | Increased |
|  |  |  | 3 vs. +7.5 | 0.9999 | No | Increased |
|  |  |  | 3 vs. +24 | 0.0002 | Yes | Increased |
|  |  |  |  |  |  |  |
|  | 200 µg/mL | 10 | 3 vs. +1.5 | 0.9999 | No | Increased |
|  |  |  | 3 vs. +4.5 | 0.9999 | No | Increased |
|  |  |  | 3 vs. +7.5 | 0.9428 | No | Increased |
|  |  |  | 3 vs. +24 | 0.0004 | Yes | Increased |
|  |  |  |  |  |  |  |
|  | 200 µg/mL | 100 | 3 vs. +1.5 | 0.9998 | No | Increased |
|  |  |  | 3 vs. +4.5 | 0.9852 | No | Increased |
|  |  |  | 3 vs. +7.5 | 0.8819 | No | Increased |
|  |  |  | 3 vs. +24 | 0.0067 | Yes | Increased |
