# Supplementary material for: Effect of Different Antibiotic Chemotherapies on Pseudomonas aeruginosa Infection In Vitro of Primary Human Corneal Fibroblast Cells
Source: Front Microbiol. 2017 Aug 22;8:1614. doi: 10.3389/fmicb.2017.01614 (PMC5572282; doi:10.3389/fmicb.2017.01614)
Supplement: Supplementary file 7 [file Table_3.DOCX]

**Supplementary Table 3. One-way ANOVA with Dunnett’s múltiple comparison test for the data shown in Figure 5.**

| **Antibiotic** | **Concentration** | **Compartment** | **Test (h)** | **Adjusted P Value** | **Significance** | **CFU numbers** |
| --- | --- | --- | --- | --- | --- | --- |
| **Ciprofloxacin** | 50µg/mL | Intracellular | 24 vs. +3 | 0.9999 | No | Similar |
|  |  |  | 24 vs. +6 | 0.9999 | No | Increased |
|  |  |  | 24 vs. +9 | 0.0008 | Yes | Increased |
|  |  |  | 24 vs. +24 | 0.0007 | Yes | Increased |
|  |  |  | 24 vs. +48 | 0.0001 | Yes | Increased |
|  |  | Extracellular | 24 vs. +3 | 0.9999 | No | Similar |
|  |  |  | 24 vs. +6 | 0.9999 | No | Similar |
|  |  |  | 24 vs. +9 | 0.9999 | No | Similar |
|  |  |  | 24 vs. +24 | 0.007 | Yes | Increased |
|  |  |  | 24 vs. +48 | 0.0001 | Yes | Increased |
|  |  |  |  |  |  |  |
|  | 200µg/mL | Intracellular | 24 vs. +3 | 0.9999 | No | Similar |
|  |  |  | 24 vs. +6 | 0.9999 | No | Increased |
|  |  |  | 24 vs. +9 | 0.9999 | No | Increased |
|  |  |  | 24 vs. +24 | 0.0075 | Yes | Increased |
|  |  |  | 24 vs. +48 | 0.0001 | Yes | Increased |
|  |  | Extracellular | 24 vs. +3 | 0.9999 | No | Similar |
|  |  |  | 24 vs. +6 | 0.9999 | No | Similar |
|  |  |  | 24 vs. +9 | 0.9999 | No | Similar |
|  |  |  | 24 vs. +24 | 0.9999 | No | Similar |
|  |  |  | 24 vs. +48 | 0.0001 | Yes | Increased |
|  |  |  |  |  |  |  |
| **Antibiotic** | **Concentration** | **Compartment** | **Test (h)** | **Adjusted P Value** | **Significance** | **CFU numbers** |
| **Levofloxacin** | 50µg/mL | Intracellular | 24 vs. +3 | 0.9999 | No | Similar |
|  |  |  | 24 vs. +6 | 0.049 | Yes | Increased |
|  |  |  | 24 vs. +9 | 0.033 | Yes | Increased |
|  |  |  | 24 vs. +24 | 0.009 | Yes | Increased |
|  |  |  | 24 vs. +48 | 0.0001 | Yes | Increased |
|  |  | Extracellular | 24 vs. +3 | 0.9999 | No | Similar |
|  |  |  | 24 vs. +6 | 0.9999 | No | Similar |
|  |  |  | 24 vs. +9 | 0.9999 | No | Similar |
|  |  |  | 24 vs. +24 | 0.0329 | Yes | Increased |
|  |  |  | 24 vs. +48 | 0.0001 | Yes | Increased |
|  |  |  |  |  |  |  |
|  | 200µg/mL | Intracellular | 24 vs. +3 | 0.9999 | No | Similar |
|  |  |  | 24 vs. +6 | 0.879 | No | Increased |
|  |  |  | 24 vs. +9 | 0.048 | Yes | Increased |
|  |  |  | 24 vs. +24 | 0.0018 | Yes | Increased |
|  |  |  | 24 vs. +48 | 0.0001 | Yes | Increased |
|  |  | Extracellular | 24 vs. +3 | 0.9999 | No | Similar |
|  |  |  | 24 vs. +6 | 0.9999 | No | Similar |
|  |  |  | 24 vs. +9 | 0.9999 | No | Similar |
|  |  |  | 24 vs. +24 | 0.9999 | No | Similar |
|  |  |  | 24 vs. +48 | 0.0001 | Yes | Increased |
|  |  |  |  |  |  |  |
|  |  |  |  |  |  |  |
|  |  |  |  |  |  |  |
| **Antibiotic** | **Concentration** | **Compartment** | **Test (h)** | **Adjusted P Value** | **Significance** | **CFU numbers** |
| **Ofloxacin** | 50µg/mL | Intracellular | 24 vs. +3 | 0.9999 | No | Similar |
|  |  |  | 24 vs. +6 | 0.048 | Yes | Increased |
|  |  |  | 24 vs. +9 | 0.0419 | Yes | Increased |
|  |  |  | 24 vs. +24 | 0.038 | Yes | Increased |
|  |  |  | 24 vs. +48 | 0.0001 | Yes | Increased |
|  |  | Extracellular | 24 vs. +3 | 0.9999 | No | Similar |
|  |  |  | 24 vs. +6 | 0.9999 | No | Similar |
|  |  |  | 24 vs. +9 | 0.9999 | No | Similar |
|  |  |  | 24 vs. +24 | 0.031 | Yes | Increased |
|  |  |  | 24 vs. +48 | 0.0003 | Yes | Increased |
|  |  |  |  |  |  |  |
|  | 200µg/mL | Intracellular | 24 vs. +3 | 0.9999 | No | Similar |
|  |  |  | 24 vs. +6 | 0.038 | Yes | Increased |
|  |  |  | 24 vs. +9 | 0.008 | Yes | Increased |
|  |  |  | 24 vs. +24 | 0.0079 | Yes | Increased |
|  |  |  | 24 vs. +48 | 0.0001 | Yes | Increased |
|  |  | Extracellular | 24 vs. +3 | 0.9999 | No | Similar |
|  |  |  | 24 vs. +6 | 0.9999 | No | Similar |
|  |  |  | 24 vs. +9 | 0.9999 | No | Similar |
|  |  |  | 24 vs. +24 | 0.9999 | No | Similar |
|  |  |  | 24 vs. +48 | 0.0001 | Yes | Increased |
|  |  |  |  |  |  |  |
| **Antibiotic** | **Concentration** | **Compartment** | **Test (h)** | **Adjusted P Value** | **Significance** |  |
| **Polymyxin B** | 50µg/mL | Intracellular | 24 vs. +3 | 0.9999 | No | Similar |
|  |  |  | 24 vs. +6 | 0.9999 | No | Similar |
|  |  |  | 24 vs. +9 | 0.9999 | No | Similar |
|  |  |  | 24 vs. +24 | 0.0001 | Yes | Increased |
|  |  |  | 24 vs. +48 | 0.0001 | Yes | Increased |
|  |  | Extracellular | 24 vs. +3 | 0.9999 | No | Similar |
|  |  |  | 24 vs. +6 | 0.047 | Yes | Increased |
|  |  |  | 24 vs. +9 | 0.0373 | Yes | Increased |
|  |  |  | 24 vs. +24 | 0.0001 | Yes | Increased |
|  |  |  | 24 vs. +48 | 0.0001 | Yes | Increased |
|  | 200µg/mL | Intracellular | 24 vs. +3 | 0.9999 | No | Similar |
|  |  |  | 24 vs. +6 | 0.9999 | No | Similar |
|  |  |  | 24 vs. +9 | 0.9999 | No | Similar |
|  |  |  | 24 vs. +24 | 0.9999 | No | Similar |
|  |  |  | 24 vs. +48 | 0.0001 | Yes | Increased |
|  |  | Extracellular | 24 vs. +3 | 0.9999 | No | Similar |
|  |  |  | 24 vs. +6 | 0.9999 | No | Similar |
|  |  |  | 24 vs. +9 | 0.9999 | No | Similar |
|  |  |  | 24 vs. +24 | 0.9999 | No | Similar |
|  |  |  | 24 vs. +48 | 0.0001 | Yes | Increased |
|  |  |  |  |  |  |  |
| **Antibiotic** | **Concentration** | **Compartment** | **Test (h)** | **Adjusted P Value** | **Significance** | **CFU numbers** |
| **Gentamicin** | 50µg/mL | Intracellular | 24 vs. +3 | 0.9999 | No | Similar |
|  |  |  | 24 vs. +6 | 0.9999 | No | Similar |
|  |  |  | 24 vs. +9 | 0.9999 | No | Similar |
|  |  |  | 24 vs. +24 | 0.0001 | Yes | Increased |
|  |  |  | 24 vs. +48 | 0.0001 | Yes | Increased |
|  |  | Extracellular | 24 vs. +3 | 0.9999 | No | Increased |
|  |  |  | 24 vs. +6 | 0.0355 | Yes | Increased |
|  |  |  | 24 vs. +9 | 0.046 | Yes | Increased |
|  |  |  | 24 vs. +24 | 0.0001 | Yes | Increased |
|  |  |  | 24 vs. +48 | 0.0001 | Yes | Increased |
|  |  |  |  |  |  |  |
|  | 200µg/mL | Intracellular | 24 vs. +3 | 0.9999 | No | Similar |
|  |  |  | 24 vs. +6 | 0.9999 | No | Similar |
|  |  |  | 24 vs. +9 | 0.9999 | No | Similar |
|  |  |  | 24 vs. +24 | 0.0001 | Yes | Increased |
|  |  |  | 24 vs. +48 | 0.0001 | Yes | Increased |
|  |  | Extracellular | 24 vs. +3 | 0.9999 | No | Similar |
|  |  |  | 24 vs. +6 | 0.0346 | Yes | Increased |
|  |  |  | 24 vs. +9 | 0.0299 | Yes | Increased |
|  |  |  | 24 vs. +24 | 0.0001 | Yes | Increased |
|  |  |  | 24 vs. +48 | 0.0001 | Yes | Increased |
