# Supplementary material for: Effect of Different Antibiotic Chemotherapies on Pseudomonas aeruginosa Infection In Vitro of Primary Human Corneal Fibroblast Cells
Source: Front Microbiol. 2017 Aug 22;8:1614. doi: 10.3389/fmicb.2017.01614 (PMC5572282; doi:10.3389/fmicb.2017.01614)
Supplement: Supplementary file 8 [file Table_4.DOCX]

**Supplementary Table 4. One-way ANOVA with Dunnett’s múltiple comparison test for the cytokine data shown in Figure 6.**

| **MOI** | **Antibiotic concentration** | **Test condition** | **Adjusted P Value** | **Significance** | **IL-1β levels** |
| --- | --- | --- | --- | --- | --- |
| 0 | 50µg/mL | CIP vs no antibiotic | 0.2369 | No | Similar |
|  |  | LVX vs no antibiotic | 0.3028 | No | Similar |
|  |  | PMB vs no antibiotic | 0.0001 | Yes | Increased |
|  |  | GEN vs no antibiotic | 0.0583 | No | Similar |
|  |  | OFX vs no antibiotic | 0.0001 | Yes | Increased |
|  |  | CXM vs no antibiotic | 0.0143 | Yes | Increased |
|  |  | CHL vs no antibiotic | 0.0110 | Yes | Increased |
|  | 200µg/mL | CIP vs no antibiotic | 0.9999 | No | Similar |
|  |  | LVX vs no antibiotic | 0.6274 | No | Similar |
|  |  | PMB vs no antibiotic | 0.0006 | Yes | Increased |
|  |  | GEN vs no antibiotic | 0.9999 | No | Similar |
|  |  | OFX vs no antibiotic | 0.0096 | Yes | Increased |
|  |  | CXM vs no antibiotic | 0.9999 | No | Similar |
|  |  | CHL vs no antibiotic | 0.9057 | No | Similar |
| 1 | 50µg/mL | CIP vs no antibiotic | 0.0001 | Yes | Reduced |
|  |  | LVX vs no antibiotic | 0.0001 | Yes | Reduced |
|  |  | PMB vs no antibiotic | 0.0001 | Yes | Reduced |
|  |  | GEN vs no antibiotic | 0.0001 | Yes | Reduced |
|  |  | OFX vs no antibiotic | 0.0001 | Yes | Reduced |
|  |  | CXM vs no antibiotic | 0.0001 | Yes | Reduced |
|  |  | CHL vs no antibiotic | 0.0012 | Yes | Reduced |
|  | 200µg/mL | CIP vs no antibiotic | 0.0004 | Yes | Reduced |
|  |  | LVX vs no antibiotic | 0.0020 | Yes | Reduced |
|  |  | PMB vs no antibiotic | 0.0777 | No | Similar |
|  |  | GEN vs no antibiotic | 0.0005 | Yes | Reduced |
|  |  | OFX vs no antibiotic | 0.0125 | Yes | Reduced |
|  |  | CXM vs no antibiotic | 0.0006 | Yes | Reduced |
|  |  | CHL vs no antibiotic | 0.0107 | Yes | Reduced |
| 10 | 50µg/mL | CIP vs no antibiotic | 0.0053 | Yes | Reduced |
|  |  | LVX vs no antibiotic | 0.0025 | Yes | Reduced |
|  |  | PMB vs no antibiotic | 0.4026 | No | Similar |
|  |  | GEN vs no antibiotic | 0.0017 | Yes | Reduced |
|  |  | OFX vs no antibiotic | 0.0024 | Yes | Reduced |
|  |  | CXM vs no antibiotic | 0.3435 | No | Similar |
|  |  | CHL vs no antibiotic | 0.1416 | No | Similar |
|  | 200µg/mL | CIP vs no antibiotic | 0.0315 | Yes | Reduced |
|  |  | LVX vs no antibiotic | 0.0700 | No | Similar |
|  |  | PMB vs no antibiotic | 0.0001 | Yes | Increased |
|  |  | GEN vs no antibiotic | 0.0335 | Yes | Reduced |
|  |  | OFX vs no antibiotic | 0.7905 | No | Similar |
|  |  | CXM vs no antibiotic | 0.5196 | No | Similar |
|  |  | CHL vs no antibiotic | 0.4197 | No | Similar |
| 100 | 50µg/mL | CIP vs no antibiotic | 0.6391 | No | Similar |
|  |  | LVX vs no antibiotic | 0.6440 | No | Similar |
|  |  | PMB vs no antibiotic | 0.3195 | No | Similar |
|  |  | GEN vs no antibiotic | 0.1120 | No | Similar |
|  |  | OFX vs no antibiotic | 0.2803 | No | Similar |
|  |  | CXM vs no antibiotic | 0.9999 | No | Similar |
|  |  | CHL vs no antibiotic | 0.9999 | No | Similar |
|  | 200µg/mL | CIP vs no antibiotic | 0.0036 | Yes | Reduced |
|  |  | LVX vs no antibiotic | 0.1761 | No | Similar |
|  |  | PMB vs no antibiotic | 0.9906 | No | Similar |
|  |  | GEN vs no antibiotic | 0.0013 | Yes | Reduced |
|  |  | OFX vs no antibiotic | 0.0009 | Yes | Increased |
|  |  | CXM vs no antibiotic | 0.0830 | No | Similar |
|  |  | CHL vs no antibiotic | 0.4458 | No | Similar |
